# The World Health Organization Fetal Growth Charts: A Multinational Longitudinal Study of Ultrasound Biometric Measurements and Estimated Fetal Weight

**DOI:** 10.1371/journal.pmed.1002220

**Published:** 2017-01-24

**Authors:** Torvid Kiserud, Gilda Piaggio, Guillermo Carroli, Mariana Widmer, José Carvalho, Lisa Neerup Jensen, Daniel Giordano, José Guilherme Cecatti, Hany Abdel Aleem, Sameera A. Talegawkar, Alexandra Benachi, Anke Diemert, Antoinette Tshefu Kitoto, Jadsada Thinkhamrop, Pisake Lumbiganon, Ann Tabor, Alka Kriplani, Rogelio Gonzalez Perez, Kurt Hecher, Mark A. Hanson, A. Metin Gülmezoglu, Lawrence D. Platt

**Affiliations:** 1 Department of Obstetrics and Gynecology, Haukeland University Hospital, Bergen, Norway; 2 Department of Clinical Science, University of Bergen, Bergen, Norway; 3 Medical Statistics Department, London School of Hygiene &Tropical Medicine, London, United Kingdom; 4 Statistika Consultoria, São Paulo, Brazil; 5 Centro Rosarino de Estudios Perinatales, Rosario, Argentina; 6 Department of Reproductive Health and Research, UNDP/UNFPA/UNICEF/WHO/World Bank Special Programme of Research, Development and Research Training in Human Reproduction, World Health Organization, Geneva, Switzerland; 7 Center of Fetal Medicine, Department of Obstetrics, Rigshospitalet, Copenhagen University, Copenhagen, Denmark; 8 Department of Obstetrics and Gynecology, School of Medical Sciences, University of Campinas, Campinas, Brazil; 9 Department of Obstetrics and Gynecology, Faculty of Medicine, Assiut University, Assiut, Egypt; 10 Department of Exercise and Nutrition Sciences, Milken Institute School of Public Health, George Washington University, Washington, District of Columbia, United States of America; 11 Service de Gynecologie Obstetrique, Hôpital Antoine-Béclère, AP-HP, Université Paris Sud, Clamart, France; 12 Department for Obstetrics and Fetal Medicine, University Medical Center Hamburg-Eppendorf, Hamburg, Germany; 13 École de Santé Publique, Faculté de Medecine, Université de Kinshasa, Kinshasa, Democratic Republic of the Congo; 14 Department of Obstetrics and Gynecology, Faculty of Medicine, Khon Kaen University, Khon Kaen, Thailand; 15 Department of Obstetrics and Gynecology, All India Institute of Medical Sciences, New Delhi, India; 16 División de Obstetricia y Ginecología, Escuela de Medicina, Pontificia Universidad Católica de Chile, Santiago, Chile; 17 Institute of Developmental Sciences, University of Southampton, Southampton, United Kingdom; 18 David Geffen School of Medicine, University of California, Los Angeles, Los Angeles, California, United States of America; 19 Center for Fetal Medicine and Women’s Ultrasound, Los Angeles, California, United States of America; University of Manchester, UNITED KINGDOM

## Abstract

**Background:**

Perinatal mortality and morbidity continue to be major global health challenges strongly associated with prematurity and reduced fetal growth, an issue of further interest given the mounting evidence that fetal growth in general is linked to degrees of risk of common noncommunicable diseases in adulthood. Against this background, WHO made it a high priority to provide the present fetal growth charts for estimated fetal weight (EFW) and common ultrasound biometric measurements intended for worldwide use.

**Methods and Findings:**

We conducted a multinational prospective observational longitudinal study of fetal growth in low-risk singleton pregnancies of women of high or middle socioeconomic status and without known environmental constraints on fetal growth. Centers in ten countries (Argentina, Brazil, Democratic Republic of the Congo, Denmark, Egypt, France, Germany, India, Norway, and Thailand) recruited participants who had reliable information on last menstrual period and gestational age confirmed by crown–rump length measured at 8–13 wk of gestation. Participants had anthropometric and nutritional assessments and seven scheduled ultrasound examinations during pregnancy. Fifty-two participants withdrew consent, and 1,387 participated in the study.

At study entry, median maternal age was 28 y (interquartile range [IQR] 25–31), median height was 162 cm (IQR 157–168), median weight was 61 kg (IQR 55–68), 58% of the women were nulliparous, and median daily caloric intake was 1,840 cal (IQR 1,487–2,222).

The median pregnancy duration was 39 wk (IQR 38–40) although there were significant differences between countries, the largest difference being 12 d (95% CI 8–16). The median birthweight was 3,300 g (IQR 2,980–3,615). There were differences in birthweight between countries, e.g., India had significantly smaller neonates than the other countries, even after adjusting for gestational age. Thirty-one women had a miscarriage, and three fetuses had intrauterine death.

The 8,203 sets of ultrasound measurements were scrutinized for outliers and leverage points, and those measurements taken at 14 to 40 wk were selected for analysis. A total of 7,924 sets of ultrasound measurements were analyzed by quantile regression to establish longitudinal reference intervals for fetal head circumference, biparietal diameter, humerus length, abdominal circumference, femur length and its ratio with head circumference and with biparietal diameter, and EFW. There was asymmetric distribution of growth of EFW: a slightly wider distribution among the lower percentiles during early weeks shifted to a notably expanded distribution of the higher percentiles in late pregnancy.

Male fetuses were larger than female fetuses as measured by EFW, but the disparity was smaller in the lower quantiles of the distribution (3.5%) and larger in the upper quantiles (4.5%). Maternal age and maternal height were associated with a positive effect on EFW, particularly in the lower tail of the distribution, of the order of 2% to 3% for each additional 10 y of age of the mother and 1% to 2% for each additional 10 cm of height. Maternal weight was associated with a small positive effect on EFW, especially in the higher tail of the distribution, of the order of 1.0% to 1.5% for each additional 10 kg of bodyweight of the mother. Parous women had heavier fetuses than nulliparous women, with the disparity being greater in the lower quantiles of the distribution, of the order of 1% to 1.5%, and diminishing in the upper quantiles. There were also significant differences in growth of EFW between countries. In spite of the multinational nature of the study, sample size is a limiting factor for generalization of the charts.

**Conclusions:**

This study provides WHO fetal growth charts for EFW and common ultrasound biometric measurements, and shows variation between different parts of the world.

## Abstract 2

Using data from a multinational longitudinal study, Mariana Widmer and colleagues report The World Health Organization fetal growth charts.

## Author Summary

**Why Was This Study Done?:**

- Small size at birth is associated with perinatal mortality, child morbidity, and adult health risks, all major global health challenges prioritized by the World Health Organization.
- Ultrasound estimation of fetal weight before birth is today very widely used in clinical practice, and, while essential for the identification and management of high-risk pregnancies, the current reference ranges used worldwide are largely based on single populations from a few high-income countries and are therefore of uncertain general applicability.
- WHO therefore requested new fetal growth charts based on multiple populations to be made available for general use and at the same time provide a foundation for the growing initiative to prevent noncommunicable diseases and promote a healthy life course starting before birth.

**What Did the Researchers Do and Find?:**

- In all, 1,387 healthy women with low-risk pregnancies and unconstrained nutritional and social background from ten countries in Africa, Asia, Europe, and South America were included in a longitudinal study of fetal growth.
- During pregnancy, repeated ultrasound measurements were used to establish international fetal growth charts for head and abdominal circumference, length of the thigh bone, and fetal weight, estimated using a combination of the three measurements.
- Fetal growth showed considerable natural variation, differing significantly between countries. Growth was to a small extent influenced by maternal age, height, weight, and parity, and by fetal sex.
- Similarly, birthweight varied significantly between countries, even after adjustment for differences in the length of pregnancy.

**What Do These Findings Mean?:**

- We suggest that these WHO charts for growth in estimated fetal weight are more suitable for international use than those commonly applied today. However, the differences between countries, with maternal factors, and with fetal sex mean that these growth charts may need to be adjusted for local clinical use to increase their diagnostic and predictive performance.
- The considerable variation in fetal growth and birthweight which occurs even under optimal conditions, and which is not explicable in terms of maternal and population factors, may suggest, first, that such natural variation in offspring size is a collective adaptive strategy that has proved extremely successful from an evolutionary point of view and, second, that major determinants of variation in human development before birth are still to be determined.
- Although the present study encompasses ten countries, it still represents only a small selection when the substantial anthropometric variations existing even within continents are taken into account.

## Introduction

Global mortality for infants under age 5 y halved from 90 to 43 deaths per 1,000 live births between 1990 and 2015. This is the result of a tremendous global effort to achieve the UN Millennium Development Goals [1] and the goals of the UN Secretary-General’s Every Woman Every Child initiative [2]. Neonatal mortality in the first 28 d declined (by 47%) from 5.0 to 2.6 million deaths annually over this period. Unfortunately, inequality between countries persists, with 98% of neonatal deaths occurring in low- and middle-income countries [3]. Importantly, more than 60% of such deaths are associated with low birthweight due to intrauterine growth restriction or preterm birth or both [4,5]. Ultrasound imaging has become an essential tool for assuring correct gestational age and for fetal size assessment, increasingly so even in societies with restricted resources. Correspondingly, evidence is emerging at the population level that use of ultrasound biometry increases the rate of detection of fetal growth restriction and the identification of those at increased risk of neonatal morbidity [6].

Birthweight, closely linked to fetal growth, is also a marker of risks for noncommunicable diseases in adult life, with cardiovascular diseases, type II diabetes, and obesity being the most prominent [7,8]. While the birthweight gradient across the entire population reflects the distribution of degrees of such risk, it is increasingly evident that it is the developing physiology associated with fetal growth, rather than birthweight per se, that conditions cardiovascular, metabolic, endocrine, and neural functions for the life course, and thus long-term health and disease risks [9]. For this reason, fetal growth data and aspects of intrauterine development need to be included as an important part of an early-life noncommunicable disease prevention initiative, as this targets the time when the effect of an intervention is greatest [10].

A meeting of experts convened by WHO in 2002 reviewed current knowledge on birthweight as a health outcome and identified a need for research to develop fetal growth charts for international use [11]. In 2006, WHO published the multicenter WHO Child Growth Standards [12] using a prescriptive concept that assumes that, under optimal socioeconomic and nutritional conditions, all children follow one growth standard, regardless of ethnic background. Some support for this concept was drawn from previous studies [13,14]. Although widely adopted, the applicability of these child growth standards has been questioned on the grounds of lack of fit to some populations [15,16], especially for the head circumference standards [17].

Recently, a large multicenter study, the Fetal Growth Longitudinal Study of the Intergrowth-21st Project [18], applied the same concept and approach to fetal growth. The study presented growth standards using ultrasound biometric measurements but did not estimate fetal weight (EFW), even though this is the single most widely used clinical assessment of fetal growth today. Another large recent study, the NICHD Fetal Growth Studies, showed significant differences in fetal growth with ethnicity, and established ethnic-specific growth charts [19]. This contradicts the prescriptive concept that one standard fits all. The study was, however, restricted to four self-reported ethnic groups of Asian, Hispanic, black, and white women in the US.

The present study is the fetal component of the WHO Multicentre Growth Reference Study, which aimed to establish growth charts for clinical use based on populations recruited from multiple countries [20].

## Methods

### Design

This was a multinational observational study approved by the WHO Research Project Review Panel (RP2) and the WHO Research Ethics Review Committee, secondarily approved by the national or local ethics review committee for each study center, and correspondingly carried out according to the Helsinki declaration on ethical principles for medical research in humans [20,21]. All women were recruited specifically for this study, gave written informed consent at inclusion, and otherwise followed their conventional antenatal care program separately from study sessions. Study measurements were revealed to the clinician when the information was thought to be of importance for the management of the pregnancy. The study protocol was published previously [20], so here we present a condensed account of the methods. The study selected participating centers from a range of ethnic and geographical settings, and intended to recruit 1,400 participants. The sample size calculation procedure was published previously [20].

### Setting

The following centers participated in the study based on the proficient use of ultrasonography: Centro Rosarino de Estudios Perinatales, Rosario, Argentina; University of Campinas, Campinas, Brazil; University of Kinshasa, Kinshasa, Democratic Republic of the Congo (D. R. Congo); Rigshospitalet, Copenhagen University, Copenhagen, Denmark; Assiut University, Assiut, Egypt; Hôpital Antoine Béclère, Paris, France; University Medical Center, Hamburg-Eppendorf, Germany; All India Institute of Medical Sciences, New Delhi, India; Haukeland University Hospital, Bergen, Norway; and Khon Kaen University, Khon Kaen, Thailand.

### Participants

Participants without known health, environmental, and/or socioeconomic constraints were invited to participate in the study. Further inclusion criteria were used: living at an altitude lower than 1,500 m and near the study area (intended to promote compliance for the duration of the study and any possible follow-up studies); age ≥ 18 y and ≤ 40 y; body mass index (BMI) 18–30 kg/m^2^; singleton pregnancy; gestational age at entry between gestational week 8+0 d and 12+6 d according to reliable information on last menstrual period (LMP) and confirmed by ultrasound measurement of fetal crown–rump length; no history of chronic health problems; no long-term medication (including fertility treatment); no environmental or economic constraints likely to impede fetal growth; not smoking currently or in the previous 6 mo; no history of recurrent miscarriages; no previous preterm delivery (<37 wk) or birthweight < 2,500 g; and no evidence in the present pregnancy of congenital disease or fetal anomaly at study entry. Fetal anomalies detected during pregnancy or at birth were noted and verified postnatally. Pregnancies in which small-for-gestation-age fetuses were observed or intrauterine growth restriction was suspected were also noted. All mothers recruited were followed up until the end of the study, apart from those withdrawing consent.

### Study Procedures

Women in the first trimester (before week 12+6 d of gestation) attending antenatal care clinics were approached by members of the study team and asked to participate. They were informed about the study objectives and procedures. Those who signed the consent form were enrolled in the study. After the ultrasound scan to assess agreement between gestational age based on LMP and that based on crown–rump length, they were scheduled for fetal biometry scans at monthly intervals.

All infants had an anthropometric assessment after delivery, including measurement of birthweight. All pregnant women in the study were asked for a 24-h dietary recall at entry into the study (and at 28 and 36 wk of gestation) [22]. Clinically relevant conditions (e.g., hypertension, preeclampsia, and diabetes) occurring during pregnancy and childbirth were noted. Otherwise, no further procedures were added to the routine antenatal care provided at the study centers.

### Gestational Age Assessment

Gestational age was confirmed by measuring the crown–rump length between gestational week 8 + 0 d and 12 + 6 d based on LMP and recorded as the average of three measurements. To acquire the crown–rump length, the midline sagittal section of the whole fetus was visualized with the fetus horizontal on the screen at 90 degrees to the angle of insonation. Gestational age was assessed by using the reference charts published by Robinson and Fleming [23]. The woman was eligible for the study provided that gestational age by crown–rump length confirmed LMP-based age within 7 d. The LMP-based age was used for the analyses.

### Ultrasound Measurements

The first visit (dating scan) was between 8 + 0 and 12 + 6 wk, and subsequent visits for fetal biometry were scheduled at approximately 4-wk (±1 wk) intervals at 14, 18, 24, 28, 32, 36, and 40 wk. All scanning appointments were arranged at the time of the dating scan and study enrollment. All participants were scanned in the lateral recumbent position.

The compulsory ultrasound measurements obtained at all visits included the following biometric parameters: biparietal diameter (BPD), head circumference (HC), abdominal circumference (AC), femur length (FL), and humerus length (HL). At each examination, all measurements were obtained three times from three separately generated ultrasound images and uploaded electronically (with the associated images) to the data management system. The median of the three measurements of each parameter was used in the analyses.

In addition, a full morphological evaluation (anomaly scan) was conducted at 18–24 wk following standard practice at each center. Fetuses diagnosed with any anomaly were managed according to local clinical guidelines. Their ultrasound measurements were included in the study, and the possible effect on the percentiles derived was evaluated. The following measurement techniques were used. BPD was measured as the outer–inner distance of the parietal bones in a cross-sectional view of the fetal head at the level of the thalami and cavum septi pellucidi or cerebral peduncles. The cerebellum was not included in the section. The measurement was obtained from an image with the midline echo as close as possible to the horizontal plane, 90 degrees to the ultrasound beam. HC was obtained from the same image as BPD as follows: calipers were placed on the outer borders of the occipital and frontal edges of the bone at the point of the midline of the skull, and the ellipse facility was used to follow the outer perimeter of the skull to calculate HC. AC was measured in the transverse section of the fetal abdomen that was as close as possible to circular and that included the stomach and the junction of the umbilical vein and portal sinus. The anteroposterior and transverse diameters were then measured with calipers placed on the outer borders of the body outline. The anteroposterior diameter was measured from the spine to the anterior abdominal wall, and the transverse diameter at a right angle to the anteroposterior diameter. The ellipse facility was used to calculate AC as outlined above. FL was measured from an image of the full femoral shaft in a plane close to 90 degrees to the ultrasound beam. The distal femoral epiphysis was excluded. Similarly, HL was measured from an image of the full humeral shaft in a plane close to 90 degrees to the ultrasound beam.

The participating centers used identical ultrasound machines during the project (Voluson Expert E8, General Electric, Kretz Ultrasound, Zipf, Austria) equipped with two curvilinear transabdominal transducers (4–8 MHz and 1–5 MHz) and a transvaginal transducer (6–12 MHz), observing that the energy output was set so that thermal index (TI) was <1.0. The TI was automatically recorded and transmitted to the web-based data management system by the ultrasound machine.

Measurement results were stored electronically, with the images together with all information collected from the mother and the perinatal outcomes. EFW was calculated by including HC, AC, and FL in Hadlock et al.’s third formula [24]. To facilitate assessment of relative fetal head size and growth, the ratios FL/HC and FL/BPD were established.

### Training and Quality Assurance

The choice of participating centers was based on their proficient use of ultrasound by experienced sonographers. The sonographers participating in the study received specific training for the study and were certified as proficient under the supervision of a qualified instructor, according to a standard protocol. All the ultrasound operators had their scans assessed for quality during their early period in the project. Instruments and techniques used in all centers were standardized, i.e., equipment and training were provided to each of the measurement teams.

### Maternal Anthropometric and Nutritional Assessment and Birthweight

Weight wearing light clothing was measured using a beam balance with nondetachable weights and recorded to the nearest 0.1 kg. Height of the mother was measured in the standing position using a stadiometer and recorded to the nearest millimeter. If the reading fell between two values, the lower was recorded.

The 24-h diet recall assessment was carried out by a specifically trained nutritionist or nurse who asked the study participant about food and beverages consumed during the previous 24 h [22]. Further details are available elsewhere [20]. Birthweight was assessed at delivery, and neonatal morphometry carried out within 24 h according to the protocol [20].

### Data Management

Data were collected via a web-based data management system developed by Centro Rosarino de Estudios Perinatales, Rosario, Argentina. All data (clinical, anthropometric, nutritional, and fetal biometry measurements plus 2-D/3-D images) were stored in a central server compliant with good clinical practice. Data transmission was encrypted to assure data integrity and patient confidentiality. Access to the web system was password protected, and only authorized users had access. Data changes were documented by a complete audit trail record kept automatically by the web system (recording when, by whom, and why data were changed). Data entered into the web system were checked by the coordinating unit at Centro Rosarino de Estudios Perinatales for completeness, accuracy, reliability, and consistent intended performance. Different kinds of validation procedures were carried out (checking missing values and outliers, cross-checks, cross-time verifications among scanning appointments, and protocol compliance). Measurements and 2-D/3-D images corresponding to fetal biometry had special processing. In collaboration with General Electric Healthcare, Germany, ViewPoint software was installed at all participating centers, allowing a standard interface/procedure for scans and an automatic transfer of fetal biometry measurements/images to the web-based system. Thus, all fetal biometry measurements considered by the protocol were automatically transferred instead of being entered manually (except for D. R. Congo; there, a complete checking of values was done by the comparison of images and values entered into the web-based system). The above mentioned web-based system and procedures have been used in five previous HRP (UNDP/UNFPA/UNICEF/WHO/World Bank Special Programme of Research, Development and Research Training in Human Reproduction)/WHO multicenter studies and are proven to be efficient and compliant with HRP/WHO Standard Operating Procedures as well as with Title 21 CFR Part 11 of the Code of Federal Regulations, which deals with United States Food and Drug Administration guidelines on electronic records.

### Adjustments of Analyses Compared with the Protocol and Justifications

Compared with the original protocol [20], the following aspects of the study were adjusted. Reliable information on LMP (confirmed by a measurement of crown–rump length), rather than ultrasound measured crown–rump length alone, was used as the basis for gestational age calculation for the following reasons: there is no evidence that ultrasound dating more accurately determines gestational age than a reliable LMP confirmed by crown–rump length; reliable LMP is the basis for establishing crown–rump length charts for dating; crown–rump length dating translates natural variation of size into variation of gestational age, which is not desirable for a study of growth; and LMP, not crown–rump length, is the accessible, low-cost method for gestational age assessment for all women in the world, and for the low-income areas usually the only one.

The sample size calculation was based on the assumption of normality for the distribution of ultrasound measurements. However, we used quantile regression, which calculates quantiles (i.e., percentiles) directly from the observed measurements without making assumptions about the distribution.

Maternal and fetal conditions occurring during pregnancy were not excluded from the analysis. The rationale for this was that the reference intervals of this study are intended primarily for clinical use and therefore should reflect the population for which they are intended as closely as possible. The pregnancy conditions (e.g., complications) that the study population experienced are those common to low-risk pregnancies around the world. Likewise, excluding all neonates below the 10th percentile of birthweight, as suggested in the protocol [20], would by definition remove the 10% of the participants at the bottom of the range (the vast majority being healthy in this low-risk cohort) and cause a corresponding distortion of the new growth charts, i.e., a substantial upward shift of all the lowest percentiles (10, 5, 2.5, and 1) in the direction of supernormal.

Given the plethora of measurements, we prioritized clinical usefulness in the analyses and results presented here (e.g., EFW and common biometric measurements) and left the following for secondary studies and publications: transverse cerebellar diameter, fetal foot length, 3-D ultrasound acquisitions, maternal anthropometric measurements except height and weight, the second and third sets of dietary 24-h-recall data (at 28 and 36 wk of gestation), and newborn anthropometric measurements except birthweight.

### Data Analysis and Statistical Methods

Descriptive statistics were calculated for the women’s characteristics at study entry, for mode of delivery, for birth events, and for fetal, neonatal, and maternal conditions, by country and overall. Protocol compliance was evaluated by comparing the dates of the windows of gestational age defined in the protocol with the dates of actual measurements.

The ultrasound measurements were used to estimate reference curves for individual parameters (BPD, HC, AC, FL, HL, FL/HC, FL/BPD) and EFW based on Hadlock et al.’s formula 3 [24]. Reference curves were fitted using quantile regression for reference models, as described by Wei et al. [25] from the work of Koenker [26,27].

The development of reference curves has up to now in general used parametric models, based on assumptions about distribution and on transformation of the observations to normal distributions. Advances brought by computer power and by the work of Koenker and others have made it possible to estimate the distributions directly by estimating their quantiles. Quantile regression is now a well-established technique [26,27], and statistical software is available to fit quantile regression models. Quantile regression fits a function to each chosen quantile using linear programming and has the advantage of not imposing any distributional assumptions. The asymmetry and kurtosis of the fitted distributions may thus assume any form dictated by the data, even changing with gestational age. In addition, quantile regression is more robust against the influence of outliers in the data. The flexibility of the fitting and the fact that any inference drawn is entirely data-driven led us to choose quantile regression as the method for the construction of reference curves.

The estimated quantiles were smoothed by polynomial functions of gestational age. Full models fitted a polynomial on gestational age for each country by including interaction terms between gestational age polynomial and country. Additive terms were included for other covariates.

The models were checked by the residual analysis produced by the software. Hypotheses on the overall importance of covariates were formally tested using likelihood ratio or Wald chi-square tests. In addition, visual inspection of quantile profilers was used to assess the relevance of each covariate in explaining the variation. To compare the distributions of the different countries with the overall distribution, we used quantile–quantile plots. We calculated 95% confidence intervals for the difference between country and global EFW percentiles for particular gestational ages, using the result that the parameter estimates from quantile regression were asymptotically normally distributed [28].

Logarithms of ultrasound parameters and EFW were used for the fitting. This was done only to achieve better numerical accuracy and faster convergence of the fitting algorithm. After the fitting, the results were retransformed to the original scale. To describe growth asymmetry, we used the Bowley coefficient of asymmetry [29], based on differences of semi-quartile ranges relative to the quartile range, for the gestational ages 15 and 40 wk.

Data were analyzed using SAS Software version 9.4 (SAS Institute, Cary, North Carolina, US) and JMP Pro 12 (SAS Institute, Cary, North Carolina, US).

## Results

### Participants

A total of 1,439 women were enrolled between October 2009 and September 2014, with data collection being completed with the last childbirth in April 2015. Of these, 52 (3.6%) withdrew consent, leaving 1,387 women and their fetuses participating in the study. Table 1 shows the numbers of women recruited, those withdrawing consent, those lost to follow-up, and those having miscarriages or intrauterine deaths, by country. Among women lost to follow-up and with miscarriage or intrauterine death, 10 and 15, respectively, did not contribute ultrasound information. All women other than those withdrawing consent were included in the growth curve analyses if they contributed ultrasound information, with the number in this analysis being 1,362.

**Table 1:** Number of women recruited to the study by country, with withdrawals and discontinuations.

Country	Number of Women Recruited	Consent Withdrawal		Discontinuation			
				Lost to Follow-Up		Miscarriage/Intrauterine Death*	
		*n*	Percent	*n*	Percent	*n*	Percent
Argentina	143	0	0.0	2	1.4	1	0.7
Brazil	157	4	2.5	2	1.3	3	1.9
D. R. Congo	157	15	9.6	6	3.8	10	6.4
Denmark	142	2	1.4	3	2.1	1	0.7
Egypt	180	25	13.9	11	6.1	9	5.0
France	109	1	0.9	9	8.3	2	1.8
Germany	141	0	0.0	2	1.4	0	0.0
India	146	0	0.0	7	4.8	3	2.1
Norway	140	2	1.4	1	0.7	1	0.7
Thailand	124	3	2.4	3	2.4	4	3.2
**Total**	**1,439**	**52**	**3.6**	**46**	**3.2**	**34**	**2.4**

*Two medical abortions, 29 miscarriages, and three intrauterine deaths.

D. R. Congo, Democratic Republic of the Congo.

### Population Characteristics

Statistics for participating women’s characteristics, their daily caloric intake, and ethnicity are presented in Table 2. Median age at study entry was 28 y but varied between 24 y (Argentina and Egypt) and 32 y (France). Median maternal height ranged from 155 cm (India) to 169 cm (Germany), and weight from 54 kg (Thailand) to 66 kg (Germany). While overall median BMI was 23.1 kg/m^2^, the median by country ranged from 21.6 kg/m^2^ in Thailand to 25.9 kg/m^2^ in Egypt. Median daily caloric intake in the study group was 1,848 calories according to the 24-h dietary recall assessment, with Thailand having the lowest median, 1,232 calories, and Egypt having the highest median, 2,094 calories. The ethnic distribution of the study group was roughly 20% African (including the peri-Mediterranean Egypt), 20% Asian, and 60% white.

**Table 2:** Characteristics of the participating women by country at study entry.

Characteristic	Statistic	Argentina (*N* = 143)	Brazil (*N* = 153)	D. R. Congo (*N* = 142)	Denmark (*N* = 140)	Egypt (*N* = 155)	France (*N* = 108)	Germany (*N* = 141)	India (*N* = 146)	Norway (*N* = 138)	Thailand (*N* = 121)	Total (*N* = 1,387)
**Age (y)**	**Missing**	0	0	0	0	0	0	0	0	0	0	0
	**Q1**	20	27	24	28	22	28	28	25	26	26	25
	**Median**	24	30	27	30	24	32	31	27	28	29	28
	**Q3**	28	33	31	32.5	28	34	33	30	30	32	31
**Weight (kg)**	**Missing**	0	0	0	1	8	0	0	0	1	1	11
	**Q1**	52	57	53	58	57	57	60	50	59	50	55
	**Median**	58	63	60	62	65	63	66	57	63	54	61
	**Q3**	64	69	66	67	75	69	72	62	71	59.5	68
**Height (cm)**	**Missing**	0	0	0	1	8	0	0	0	1	1	11
	**Q1**	153	160	157	164	155	162	165	152	165	155	157
	**Median**	157	163	162	168	159	165	169	155	168	157	163
	**Q3**	162	167	165	171	163	170	174	160	173	161	168
**BMI (kg/m**^**2**^**)**	**Missing**	0	0	0	1	8	0	0	0	1	1	11
	**Q1**	21.2	21.6	20.8	20.8	23.5	21.1	21.1	20.0	20.5	20.0	21.0
	**Median**	23.3	23.5	22.9	22.2	25.9	22.9	23.2	23.0	22.2	21.6	23.1
	**Q3**	26.3	25.8	25.6	24.1	29.0	24.5	24.9	25.3	24.9	23.9	25.4
**Total calories in 24-h dietary recall**	**Missing**	0	0	0	0	4	10	0	28	1	6	49
	**Q1**	1,666	1,441	1,460	1,584	1,747	1,489	1,674	1,514	1,558	1,004	1,487
	**Median**	1,928	1,709	2,063	1,820	2,094	1,736	1,978	1,831	1,890	1,232	1,848
	**Q3**	2,189	2,148	2,605	2,053	2,525	2,053	2,285	2,194	2,314	1,534	2,222
**Ethnicity, *n* (percent)**	**White**	143 (100.0)	146 (95.4)	0 (0.0)	140 (100.0)	0 (0.0)	100 (92.6)	136 (96.5)	0 (0.0)	137 (99.3)	0 (0.0)	802 (57.8)
	**Asian**	0 (0.0)	0 (0.0)	0 (0.0)	0 (0.0)	0 (0.0)	0 (0.0)	2 (1.4)	146 (100.0)	1 (0.7)	121 (100.0)	270 (19.5)
	**African**	0 (0.0)	7 (4.6)	142 (100.0)	0 (0.0)	133 (85.8)	8 (7.4)	3 (2.1)	0 (0.0)	0 (0.0)	0 (0.0)	293 (21.1)
	**Other**	0 (0.0)	0 (0.0)	0 (0.0)	0 (0.0)	22 (14.2)	0 (0.0)	0 (0.0)	0 (0.0)	0 (0.0)	0 (0.0)	22 (1.6)
**Parity (nulliparous *n*)**	***N***	137	153	142	139	57	108	141	138	138	121	1,274
	**Missing**	6	0	0	1	98	0	0	8	0	0	113
	***n* (percent)**	64 (46.7)	108 (70.6)	51 (35.9)	86 (61.9)	21 (36.8)	51 (47.2)	104 (73.8)	115 (83.3)	67 (48.6)	72 (59.5)	739 (58.0)

BMI, body mass index; D. R. Congo, Democratic Republic of the Congo; Q1, first quartile; Q3, third quartile.

### Perinatal Outcomes

Table 3 shows delivery information. The overall rate of spontaneous onset of birth was 67.3%, with a wide range by country: 28.5% in Brazil to 94.5% in D. R. Congo. There was an overall cesarean section rate of 32.1%, with a considerable range from 5.5% in D. R. Congo to 70.1% in Brazil. The occurrence of Apgar score < 7 at 5 min was similar in all countries, i.e., 0%–2.2%. Most of the countries had a similar distribution between female and male neonates except for Egypt, Germany, and Norway, where about 40% of neonates were female. The incidence of preterm birth varied from 3.6% in Germany to 14.7% in Egypt (*p* = 0.03 for differences among countries). It was lowest in D. R. Congo, Denmark, Germany, and Norway and highest in Egypt and India.

**Table 3:** Mode of delivery, gestational age at birth and outcomes.

Characteristic	Statistic	Argentina (*N* = 140)	Brazil (*N* = 150)	D. R. Congo (*N* = 127)	Denmark (*N* = 137)	Egypt (*N* = 140)	France (*N* = 97)	Germany (*N* = 139)	India (*N* = 139)	Norway (*N* = 136)	Thailand (*N* = 114)	All (*N* = 1,319)
**Neonatal sex (*n* female)**	***N***	140	148	127	136	132	97	139	137	131	112	1,299
	***n* (percent)**	68 (48.6)	70 (47.3)	67 (52.8)	75 (55.1)	54 (40.9)	45 (46.4)	56 (40.3)	67 (48.9)	52 (39.7)	54 (48.2)	608 (46.8)
**Apgar < 7 at 5 min**	***N***	140	147	127	135	136	97	139	138	136	113	1,308
	***n* (percent)**	1 (0.7)	1 (0.7)	1 (0.8)	1 (0.7)	3 (2.2)	0 (0.0)	1 (0.7)	1 (0.7)	2 (1.5)	0 (0.0)	11 (0.8)
**Preterm (gestational age < 37 wk)**	***N***	140	148	127	137	136	97	139	138	136	114	1,312
	***n* (percent)**	12 (8.6)	11 (7.4)	6 (4.7)	8 (5.8)	20 (14.7)	7 (7.2)	5 (3.6)	15 (10.9)	6 (4.4)	9 (7.9)	99 (7.5)
**Birthweight (g)**	***N***	140	148	127	136	117	97	139	137	136	113	1,290
	**Q1**	2,990	2,910	2,850	3,133	3,000	2,965	3,100	2,656	3,348	2,980	2,980
	**Median**	3,328	3,290	3,170	3,462	3,100	3,370	3,480	2,975	3,575	3,130	3,300
	**Q3**	3,620	3,608	3,500	3,790	3,500	3,600	3,820	3,200	3,900	3,400	3,615
**Gestational age (days)**	***N***	140	148	127	137	139	97	139	138	136	114	1,315
	**Q1**	270	268	270	272	262	273	273	265	276	267	269
	**Median**	276	273	277	282	271	279	279	270	283	271	276
	**Q3**	281	278	283	287	280	284	285	277	288	278	282
**Mode of delivery, *n* (percent)**	**Spontaneous**	91 (67.9)	41 (28.5)	120 (94.5)	105 (83.3)	64 (45.7)	80 (85.1)	82 (73.2)	84 (64.1)	113 (91.1)	58 (50.9)	838 (67.3)
	**Intrapartum CS**	30 (22.4)	33 (22.9)	6 (4.7)	7 (5.6)	16 (11.4)	8 (8.5)	24 (21.4)	20 (15.3)	9 (7.3)	26 (22.8)	179 (14.4)
	**Elective CS**	13 (9.7)	68 (47.2)	1 (0.8)	13 (10.3)	54 (38.6)	6 (6.4)	6 (5.4)	27 (20.6)	2 (1.6)	30 (26.3)	220 (17.7)
	**Vacuum**	0 (0.0)	0 (0.0)	0 (0.0)	11 (8.7)	0 (0.0)	0 (0.0)	25 (22.3)	5 (3.8)	1 (0.8)	0 (0.0)	42 (3.4)
	**Forceps**	6 (4.5)	6 (4.2)	0 (0.0)	0 (0.0)	0 (0.0)	3 (3.2)	2 (1.8)	3 (2.3)	11 (8.9)	0 (0.0)	31 (2.5)
	**Unknown**	0 (0.0)	2 (1.4)	0 (0.0)	1 (0.8)	6 (4.3)	0 (0.0)	0 (0.0)	0 (0.0)	0 (0.0)	0 (0.0)	9 (0.7)

CS, cesarean section; D. R. Congo, Democratic Republic of the Congo; Q1, first quartile; Q3, third quartile.

### Gestational Age at Birth and Birthweight

Gestational age at birth varied between countries from a median of 38 wk 4 d in India to 40 wk 3 d in Norway (*p <* 0.001 for differences among countries) (Table 3). Norway had the highest median birthweight (3,575 g), and Denmark and Germany had birthweights approximately 100 g less, while Argentina, Brazil, and France had birthweights 200 g less. There is a group of countries (D. R. Congo, Egypt, and Thailand) with birthweight a median 400 g less than that of Norway, and lastly India, with birthweight 500 g less. The differences in birthweight between countries were highly significant for all percentiles (*p <* 0.001 for all). When adjusted for gestational age at birth, the differences were still significant for all the percentiles (*p* = 0.0018 for the 5th percentile and *p <* 0.001 for the 10th, 25th, 50th, 75th, 90th, and 95th percentiles). The estimated birthweight according to neonatal sex and gestational age is shown in Table 4.

**Table 4:** Estimated birthweight percentiles for female and male neonates according to completed gestational week.

Percentile	Birthweight (g) by Gestational Age (wk)											
	Female						Male					
	37	38	39	40	41	42	37	38	39	40	41	42
**5**	1,968	2,315	2,575	2,748	2,835	2,834	2,062	2,451	2,723	2,880	2,921	2,845
**25**	2,493	2,698	2,891	3,072	3,241	3,398	2,705	2,890	3,061	3,218	3,362	3,491
**50**	2,786	2,990	3,173	3,336	3,479	3,601	2,919	3,153	3,354	3,519	3,650	3,747
**75**	2,951	3,217	3,443	3,631	3,779	3,888	3,143	3,387	3,608	3,806	3,982	4,134
**90**	3,181	3,451	3,682	3,871	4,021	4,130	3,450	3,666	3,871	4,067	4,253	4,428
**95**	3,238	3,593	3,867	4,060	4,171	4,200	3,584	3,813	4,036	4,251	4,459	4,659

### Maternal Complications and Perinatal Conditions

Conditions occurring in the mother during pregnancy are shown in Table 5, together with fetal malformations and neonatal conditions. In addition to globally experienced maternal complications such as preeclampsia, pregnancy-induced hypertension, gestational diabetes, and anemia, 42 had identified malaria. There was no maternal death. Four small-for-gestational-age fetuses were identified clinically, of which two were examined using Doppler ultrasound; none had abnormal recordings in the umbilical artery or middle cerebral artery, and all were kept in the analysis. It was registered when neonates needed transmission to the neonatal intensive care unit, commonly due to prematurity, respiratory distress syndrome, infections, or jaundice. There were three intrauterine deaths and three neonatal deaths, representing a perinatal mortality of 0.4%.

**Table 5:** Maternal complications, fetal malformations, and neonatal conditions by country.

Condition	Argentina (*N* = 143)	Brazil (*N* = 153)	D. R. Congo (*N* = 142)	Denmark (*N* = 140)	Egypt (*N* = 155)	France (*N* = 108)	Germany (*N* = 141)	India (*N* = 146)	Norway (*N* = 138)	Thailand (*N* = 121)	All (*N* = 1,387)
**Fetal malformation**^**§**^	4 (2.8)	0 (0.0)	0 (0.0)	0 (0.0)	1 (0.6)	1 (0.9)	1 (0.7)	0 (0.0)	1 (0.7)	0 (0.0)	8 (0.6)
**Neonatal condition**	19 (13.3)	12 (7.8)	7 (4.9)	10 (7.1)	4 (2.6)	2 (1.9)	9 (6.4)	8 (5.5)	3 (2.2)	9 (7.4)	83 (6.0)
Maternal complication*	24 (16.8)	10 (6.5)	42 (29.6)	4 (2.9)	3 (1.9)	8 (7.4)	7 (5.0)	23 (15.8)	6 (4.3)	10 (8.3)	137 (9.9)

Data are given as *n* (percent).

^**§**^One malformation was discovered at birth, here counted as fetal malformation. Sacrococcygeal cyst (1), Jarcho-Levin syndrome (1), clubfoot (1), polycystic kidneys (1), cardiac malformations (3), cleft palate (1).

*Preeclampsia (22), hypertension (16), gestational diabetes (32), malaria (42), anemia (19), and other (16); some participants had more than one diagnosis.

D. R. Congo, Democratic Republic of the Congo.

### Compliance with Ultrasound Scans

The median number of ultrasound scans (excluding the study entry screening scan) in all women was 6 (range 0–7). Compliance by gestational age window as defined in the protocol is presented in S1 Table, by country and for all countries combined (“Total”). Compliance for all countries combined in each gestational age window was between 89.1% and 100%; 72% of the participants had a complete set of all the scheduled scans. In addition, for each of the measurements BPD, HC, AC, FL, and HL, scans were obtained ≥2 times for at least 95% of participants.

### Thermal Index

Of the 8,372 scan sessions in the project, 115 had no scans stored and 54 belonged to women who withdrew consent, leaving 8,203 for the statistics. The median TI was 0.2, and none had TI ≥ 1.0.

### Reference Intervals for Biometric Parameters and Estimated Fetal Weight

**Fig 1:**
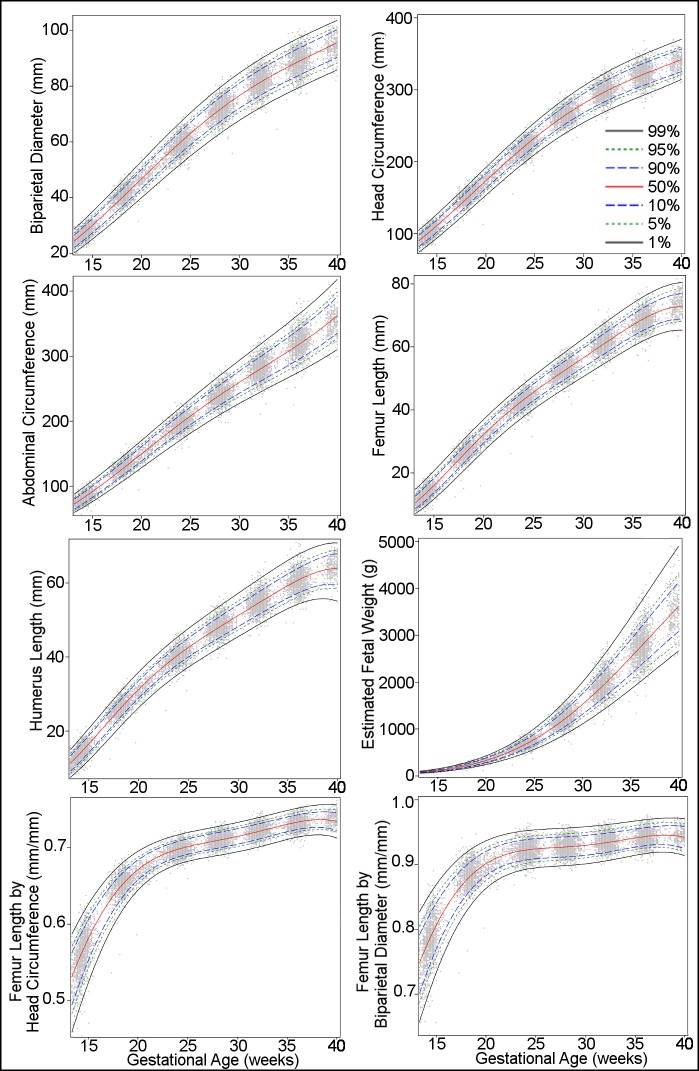
Percentiles for biparietal (outer–inner) diameter, head circumference, abdominal circumference, femur length, humerus length, estimated fetal weight, femur length/head circumference ratio, and femur length/biparietal diameter ratio during gestational weeks 14–40. The percentiles (percent) 1st, 5th, 10th, 50th, 90th, 95th, and 99th (smoothed lines) are based on quantile regression and are shown with the observed values (grey dots).

Fig 1 presents the overall growth curves for BPD, HC, AC, FL, HL, and EFW, and for the ratios FL/HC and FL/BPD, based on quantile regression. The corresponding reference values are shown in Tables 6–13 and in csv format in S1 File.

**Table 6:** Growth chart for fetal outer–inner biparietal diameter.

Gestational Age (Weeks)	Biparietal Diameter (mm) by Percentile								
	2.5	5	10	25	50	75	90	95	97.5
14	23	24	24	26	27	28	29	30	31
15	26	27	27	29	30	31	32	33	34
16	29	30	30	32	33	35	36	37	38
17	32	33	33	35	36	38	39	40	41
18	35	36	37	38	40	41	43	44	45
19	38	39	40	42	43	45	46	47	48
20	41	42	43	45	47	48	50	51	52
21	44	45	46	48	50	52	53	54	55
22	47	48	50	51	53	55	57	58	59
23	50	52	53	55	57	59	60	61	62
24	53	55	56	58	60	62	64	65	66
25	56	58	59	61	63	65	67	68	69
26	59	60	62	64	66	68	70	71	72
27	62	63	65	67	69	71	73	74	75
28	64	66	67	69	72	74	76	77	78
29	67	68	70	72	74	76	78	80	81
30	69	71	72	74	77	79	81	82	83
31	71	73	74	76	79	81	83	85	86
32	73	75	76	79	81	83	86	87	88
33	75	77	78	81	83	86	88	89	90
34	77	79	80	83	85	88	90	91	92
35	79	80	82	84	87	89	92	93	94
36	80	82	84	86	89	91	93	95	96
37	82	84	85	88	90	93	95	96	97
38	84	85	87	90	92	95	97	98	99
39	85	87	89	92	94	96	99	100	101
40	87	88	90	93	96	98	100	101	102

**Table 7:** Growth chart for fetal head circumference.

Gestational Age (Weeks)	Head Circumference (mm) by Percentile								
	2.5	5	10	25	50	75	90	95	97.5
14	86	88	91	95	100	104	107	110	112
15	97	99	102	106	111	115	119	122	124
16	108	111	114	118	123	128	132	134	137
17	120	123	126	130	135	140	144	147	149
18	132	135	138	143	148	153	157	160	162
19	145	147	150	155	161	166	170	173	175
20	157	159	163	168	173	179	183	186	188
21	169	172	175	180	186	191	196	199	201
22	181	184	187	193	198	204	209	212	214
23	193	196	199	205	210	216	221	224	227
24	204	207	211	216	222	228	233	236	239
25	215	218	222	227	233	239	245	248	251
26	225	228	232	238	244	250	256	259	262
27	234	238	242	248	254	261	267	270	273
28	243	247	251	257	264	270	277	280	283
29	251	256	260	266	273	280	286	290	293
30	259	264	268	274	281	288	295	299	302
31	266	271	275	282	289	296	303	307	311
32	273	278	282	289	296	304	311	315	318
33	279	284	289	295	303	311	318	322	326
34	285	290	295	302	309	317	324	328	332
35	291	296	300	307	315	323	330	335	338
36	296	301	306	313	321	329	336	340	344
37	302	306	311	318	326	334	341	345	349
38	307	311	315	324	332	339	347	350	354
39	313	316	320	329	337	344	352	355	359
40	319	321	325	334	342	350	357	360	363

**Table 8:** Growth chart for fetal abdominal circumference.

Gestational Age (Weeks)	Abdominal Circumference (mm) by Percentile								
	2.5	5	10	25	50	75	90	95	97.5
14	69	71	73	77	81	86	89	92	95
15	79	81	83	87	92	96	100	103	106
16	89	91	93	98	103	108	112	115	118
17	99	102	104	109	114	119	124	127	130
18	110	113	116	121	126	131	136	139	142
19	121	124	127	132	138	143	148	152	155
20	132	136	139	144	150	155	161	164	167
21	143	147	150	156	162	168	173	177	180
22	154	159	162	167	173	180	186	189	193
23	165	170	173	179	185	192	198	202	205
24	176	181	184	190	197	203	210	214	217
25	186	191	195	201	208	215	222	226	229
26	196	201	205	212	219	226	233	238	241
27	206	211	215	222	230	237	245	249	253
28	215	220	225	232	240	248	256	260	264
29	224	229	234	242	250	258	266	271	276
30	233	238	243	251	260	269	277	282	287
31	241	246	252	260	269	279	287	292	298
32	249	254	260	269	279	288	298	303	308
33	257	262	269	278	288	298	308	313	319
34	265	270	277	287	298	308	318	324	330
35	273	279	286	297	307	318	329	335	342
36	282	287	294	306	317	329	340	346	353
37	290	296	304	316	328	340	352	358	365
38	299	306	313	326	338	351	364	371	378
39	309	316	324	337	350	363	377	384	392
40	319	327	335	349	363	377	391	399	406

**Table 9:** Growth chart for fetal femur length.

Gestational Age (Weeks)	Femur Length (mm) by Percentile								
	2.5	5	10	25	50	75	90	95	97.5
14	10	10	11	12	13	14	15	16	17
15	12	13	14	15	16	17	18	19	20
16	15	16	17	18	19	20	22	22	23
17	19	19	20	21	22	24	25	26	26
18	22	22	23	24	26	27	28	29	30
19	25	26	26	28	29	30	31	32	33
20	28	29	30	31	32	33	35	35	36
21	31	32	33	34	35	36	38	38	39
22	34	35	35	37	38	39	40	41	42
23	36	37	38	39	41	42	43	44	45
24	39	40	41	42	43	45	46	47	47
25	41	42	43	44	46	47	48	49	50
26	43	44	45	46	48	49	51	51	52
27	46	46	47	49	50	52	53	54	55
28	48	48	49	51	52	54	55	56	57
29	50	50	51	53	54	56	57	58	59
30	51	52	53	55	56	58	60	60	61
31	53	54	55	57	59	60	62	63	64
32	55	56	57	59	61	62	64	65	66
33	57	58	60	61	63	65	66	67	68
34	59	60	61	63	65	67	68	69	70
35	61	62	63	65	67	69	70	71	73
36	63	64	65	67	69	70	72	73	75
37	65	66	67	68	70	72	74	75	76
38	66	67	68	70	72	74	75	77	78
39	67	68	69	70	73	75	76	78	79
40	68	68	69	70	73	75	77	78	79

**Table 10:** Growth chart for fetal humerus length.

Gestational Age (Weeks)	Humerus Length (mm) by Percentile								
	2.5	5	10	25	50	75	90	95	97.5
14	10	11	11	12	14	15	16	16	17
15	13	13	14	15	16	18	19	19	20
16	16	16	17	18	19	21	22	22	23
17	19	19	20	21	23	24	25	25	26
18	22	22	23	24	26	27	28	28	29
19	25	25	26	27	28	30	31	31	32
20	27	28	29	30	31	32	33	34	35
21	30	31	31	33	34	35	36	37	38
22	32	33	34	35	36	37	39	39	40
23	34	35	36	37	38	40	41	42	42
24	36	37	38	39	41	42	43	44	45
25	38	39	40	41	42	44	45	46	47
26	40	41	42	43	44	46	47	48	49
27	42	43	43	45	46	47	49	50	51
28	43	44	45	46	48	49	51	52	52
29	45	46	47	48	49	51	52	53	54
30	46	47	48	50	51	53	54	55	56
31	48	49	50	51	53	54	56	57	58
32	49	50	51	53	54	56	57	59	59
33	51	52	53	54	56	58	59	60	61
34	53	53	54	56	58	59	61	62	63
35	54	55	56	57	59	61	62	63	64
36	55	56	57	59	61	62	64	65	66
37	56	57	58	60	62	64	65	66	67
38	57	58	59	61	63	65	66	67	68
39	58	59	60	62	64	65	67	68	69
40	57	58	60	62	64	66	68	69	69

**Table 11:** Growth chart for estimated fetal weight regardless of fetal sex.

Gestational Age (Weeks)	Estimated Fetal Weight (g) by Percentile								
	2.5	5	10	25	50	75	90	95	97.5
14	70	73	78	83	90	98	104	109	113
15	89	93	99	106	114	124	132	138	144
16	113	117	124	133	144	155	166	174	181
17	141	146	155	166	179	193	207	217	225
18	174	181	192	206	222	239	255	268	278
19	214	223	235	252	272	292	313	328	340
20	260	271	286	307	330	355	380	399	413
21	314	327	345	370	398	428	458	481	497
22	375	392	412	443	476	512	548	575	595
23	445	465	489	525	565	608	650	682	705
24	523	548	576	618	665	715	765	803	830
25	611	641	673	723	778	836	894	938	970
26	707	743	780	838	902	971	1,038	1,087	1,125
27	813	855	898	964	1,039	1,118	1,196	1,251	1,295
28	929	977	1,026	1,102	1,189	1,279	1,368	1,429	1,481
29	1,053	1,108	1,165	1,251	1,350	1,453	1,554	1,622	1,682
30	1,185	1,247	1,313	1,410	1,523	1,640	1,753	1,828	1,897
31	1,326	1,394	1,470	1,579	1,707	1,838	1,964	2,046	2,126
32	1,473	1,548	1,635	1,757	1,901	2,047	2,187	2,276	2,367
33	1,626	1,708	1,807	1,942	2,103	2,266	2,419	2,516	2,619
34	1,785	1,872	1,985	2,134	2,312	2,492	2,659	2,764	2,880
35	1,948	2,038	2,167	2,330	2,527	2,723	2,904	3,018	3,148
36	2,113	2,205	2,352	2,531	2,745	2,959	3,153	3,277	3,422
37	2,280	2,372	2,537	2,733	2,966	3,195	3,403	3,538	3,697
38	2,446	2,536	2,723	2,935	3,186	3,432	3,652	3,799	3,973
39	2,612	2,696	2,905	3,135	3,403	3,664	3,897	4,058	4,247
40	2,775	2,849	3,084	3,333	3,617	3,892	4,135	4,312	4,515

**Table 12:** Growth chart for fetal femur length/head circumference ratio.

Gestational Age (Weeks)	Femur Length/Head Circumference Ratio by Percentile								
	2.5	5	10	25	50	75	90	95	97.5
14	0.50	0.52	0.53	0.54	0.56	0.57	0.59	0.59	0.60
15	0.54	0.55	0.56	0.57	0.59	0.60	0.61	0.62	0.62
16	0.57	0.58	0.59	0.60	0.61	0.62	0.63	0.64	0.64
17	0.60	0.60	0.61	0.62	0.63	0.64	0.65	0.65	0.66
18	0.62	0.62	0.63	0.64	0.65	0.66	0.66	0.67	0.67
19	0.64	0.64	0.65	0.65	0.66	0.67	0.68	0.68	0.68
20	0.65	0.66	0.66	0.67	0.67	0.68	0.69	0.69	0.69
21	0.66	0.67	0.67	0.68	0.68	0.69	0.69	0.70	0.70
22	0.67	0.67	0.68	0.68	0.69	0.69	0.70	0.70	0.71
23	0.68	0.68	0.68	0.69	0.69	0.70	0.70	0.71	0.71
24	0.68	0.69	0.69	0.69	0.70	0.70	0.71	0.71	0.71
25	0.69	0.69	0.69	0.70	0.70	0.71	0.71	0.71	0.72
26	0.69	0.69	0.69	0.70	0.70	0.71	0.71	0.72	0.72
27	0.69	0.69	0.70	0.70	0.71	0.71	0.72	0.72	0.72
28	0.69	0.70	0.70	0.70	0.71	0.71	0.72	0.72	0.72
29	0.70	0.70	0.70	0.71	0.71	0.72	0.72	0.72	0.73
30	0.70	0.70	0.70	0.71	0.71	0.72	0.72	0.73	0.73
31	0.70	0.70	0.71	0.71	0.72	0.72	0.73	0.73	0.73
32	0.70	0.71	0.71	0.72	0.72	0.73	0.73	0.73	0.74
33	0.71	0.71	0.71	0.72	0.72	0.73	0.73	0.74	0.74
34	0.71	0.71	0.72	0.72	0.73	0.73	0.74	0.74	0.74
35	0.71	0.72	0.72	0.73	0.73	0.74	0.74	0.74	0.75
36	0.72	0.72	0.72	0.73	0.73	0.74	0.74	0.75	0.75
37	0.72	0.72	0.73	0.73	0.74	0.74	0.74	0.75	0.75
38	0.72	0.72	0.73	0.73	0.74	0.74	0.75	0.75	0.75
39	0.72	0.72	0.73	0.73	0.74	0.74	0.75	0.75	0.75
40	0.71	0.72	0.72	0.73	0.73	0.74	0.75	0.75	0.75

**Table 13:** Growth chart for fetal femur length/biparietal diameter.

Gestational Age (Weeks)	Femur Length/Biparietal Diameter Ratio by Percentile								
	2.5	5	10	25	50	75	90	95	97.5
14	0.71	0.72	0.74	0.76	0.78	0.80	0.82	0.83	0.84
15	0.75	0.76	0.77	0.79	0.81	0.83	0.84	0.85	0.86
16	0.79	0.80	0.81	0.82	0.84	0.85	0.87	0.88	0.88
17	0.82	0.82	0.83	0.85	0.86	0.87	0.89	0.89	0.90
18	0.84	0.85	0.85	0.87	0.88	0.89	0.90	0.91	0.91
19	0.86	0.86	0.87	0.88	0.89	0.90	0.91	0.92	0.92
20	0.87	0.88	0.88	0.89	0.90	0.91	0.92	0.93	0.93
21	0.88	0.89	0.89	0.90	0.91	0.92	0.93	0.93	0.94
22	0.89	0.89	0.90	0.91	0.92	0.92	0.93	0.94	0.94
23	0.89	0.90	0.90	0.91	0.92	0.93	0.94	0.94	0.95
24	0.90	0.90	0.91	0.91	0.92	0.93	0.94	0.94	0.95
25	0.90	0.90	0.91	0.92	0.92	0.93	0.94	0.94	0.95
26	0.90	0.91	0.91	0.92	0.93	0.93	0.94	0.95	0.95
27	0.90	0.91	0.91	0.92	0.93	0.93	0.94	0.95	0.95
28	0.90	0.91	0.91	0.92	0.93	0.94	0.94	0.95	0.95
29	0.90	0.91	0.91	0.92	0.93	0.94	0.94	0.95	0.95
30	0.91	0.91	0.91	0.92	0.93	0.94	0.94	0.95	0.95
31	0.91	0.91	0.92	0.92	0.93	0.94	0.95	0.95	0.95
32	0.91	0.91	0.92	0.93	0.93	0.94	0.95	0.95	0.96
33	0.91	0.92	0.92	0.93	0.94	0.94	0.95	0.96	0.96
34	0.92	0.92	0.92	0.93	0.94	0.95	0.95	0.96	0.96
35	0.92	0.92	0.93	0.93	0.94	0.95	0.95	0.96	0.96
36	0.92	0.93	0.93	0.94	0.94	0.95	0.96	0.96	0.97
37	0.92	0.93	0.93	0.94	0.94	0.95	0.96	0.96	0.97
38	0.92	0.93	0.93	0.94	0.95	0.95	0.96	0.96	0.97
39	0.92	0.92	0.93	0.94	0.94	0.95	0.96	0.96	0.97
40	0.91	0.92	0.92	0.93	0.94	0.95	0.96	0.96	0.97

The distribution of EFW starts with a slight asymmetry to the left (i.e., lower percentiles) in early pregnancy and ends with a very noticeable right asymmetry (i.e., higher percentiles) in later pregnancy. The Bowley coefficient of asymmetry [29], based on differences of semi-quartile ranges relative to the quartile range, was −0.016 for gestational age 15 wk and +0.111 for 40 wk.

### Influence of Covariates on Growth Percentiles

#### Fetal sex

**Fig 2:**
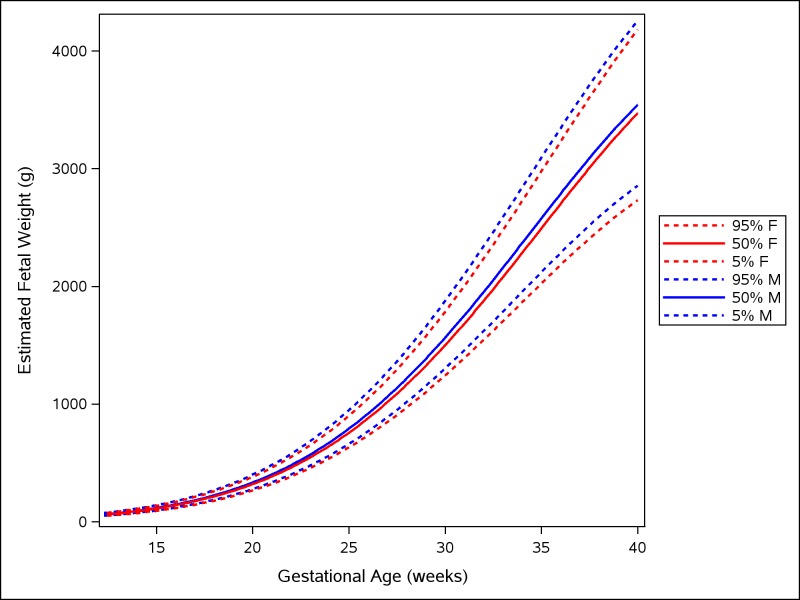
Female and male growth of estimated fetal weight during gestational weeks 14–40. The difference in growth for female (F; red) and male (M; blue) fetuses is shown by the 5th, 50th, and 95th percentiles for EFW growth. The smoothed lines are based on quantile regression that includes data from all the participating countries.

Male fetuses were larger than female fetuses as measured by EFW, but the disparity was smaller in the lower quantiles of the distribution (3.5%) and larger in the upper quantiles (4.5%) (Fig 2 and S2 Table, without adjustment for country differences). This difference in size by fetal sex was significant at the 5% level for all percentiles. EFW reference values were also established for female and male fetuses separately (Tables 14 and 15) to allow assessment customized according to fetal sex. For example, at gestational week 37, the median EFW of female fetuses is 84 g lower than that of male fetuses.

**Table 14:** Growth chart for estimated fetal weight for female fetuses.

Gestational Age (Weeks)	Female Estimated Fetal Weight (g) by Percentile						
	5	10	25	50	75	90	95
14	73	77	82	89	96	102	107
15	92	97	104	113	121	129	135
16	116	122	131	141	152	162	170
17	145	152	164	176	189	202	211
18	180	188	202	217	233	248	261
19	221	231	248	266	285	304	319
20	269	281	302	322	346	369	387
21	324	339	364	388	417	444	466
22	388	405	435	464	499	530	557
23	461	481	516	551	592	629	660
24	542	567	608	649	697	740	776
25	634	663	710	758	815	865	907
26	735	769	823	880	946	1,003	1,051
27	846	886	948	1,014	1,090	1,156	1,210
28	967	1,013	1,083	1,160	1,247	1,323	1,383
29	1,096	1,150	1,230	1,319	1,418	1,505	1,570
30	1,234	1,296	1,386	1,489	1,601	1,699	1,770
31	1,379	1,451	1,553	1,670	1,796	1,907	1,984
32	1,530	1,614	1,728	1,861	2,002	2,127	2,209
33	1,687	1,783	1,911	2,060	2,217	2,358	2,445
34	1,847	1,957	2,101	2,268	2,440	2,598	2,690
35	2,008	2,135	2,296	2,481	2,669	2,846	2,943
36	2,169	2,314	2,494	2,698	2,902	3,099	3,201
37	2,329	2,493	2,695	2,917	3,138	3,357	3,462
38	2,484	2,670	2,896	3,136	3,373	3,616	3,725
39	2,633	2,843	3,096	3,354	3,605	3,875	3,988
40	2,775	3,010	3,294	3,567	3,832	4,131	4,247

**Table 15:** Growth chart for estimated fetal weight (EFW) for male fetuses.

Gestational Age (Weeks)	Male Estimated Fetal Weight (g) by Percentile						
	5	10	25	50	75	90	95
14	75	79	84	92	99	105	109
15	96	100	107	116	126	134	139
16	121	127	136	146	158	169	175
17	152	158	170	183	197	210	219
18	188	196	210	226	243	260	271
19	232	241	258	277	298	320	333
20	282	293	314	337	362	389	405
21	341	354	380	407	436	469	489
22	408	424	454	487	522	561	586
23	484	503	539	578	619	666	695
24	570	592	635	681	730	785	818
25	666	692	742	795	853	917	956
26	772	803	860	923	990	1,063	1,109
27	888	924	989	1,063	1,141	1,224	1,276
28	1,014	1,055	1,129	1,215	1,305	1,399	1,458
29	1,149	1,197	1,281	1,379	1,482	1,587	1,654
30	1,293	1,349	1,442	1,555	1,672	1,788	1,863
31	1,445	1,509	1,613	1,741	1,874	2,000	2,085
32	1,605	1,677	1,793	1,937	2,085	2,224	2,319
33	1,770	1,852	1,980	2,140	2,306	2,456	2,562
34	1,941	2,032	2,174	2,350	2,534	2,694	2,814
35	2,114	2,217	2,372	2,565	2,767	2,938	3,072
36	2,290	2,404	2,574	2,783	3,002	3,185	3,334
37	2,466	2,591	2,777	3,001	3,238	3,432	3,598
38	2,641	2,778	2,981	3,218	3,472	3,676	3,863
39	2,813	2,962	3,183	3,432	3,701	3,916	4,125
40	2,981	3,142	3,382	3,639	3,923	4,149	4,383

#### Country

**Fig 3:**
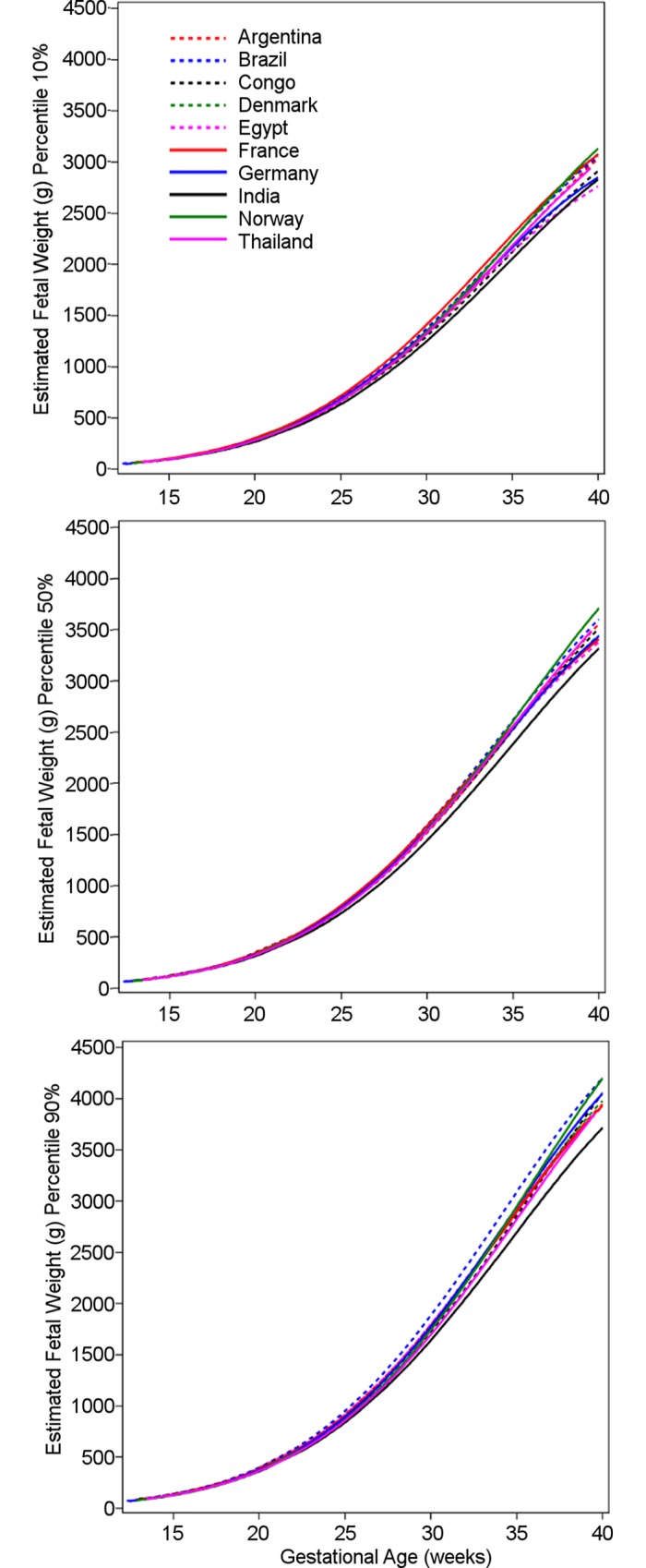
Influence of country on estimated fetal weight. The 10th, 50th, and 90th percentiles for estimated fetal weight in grams for the ten participating countries, with variation due to country becoming more obvious towards the end of gestation. Congo, Democratic Republic of the Congo.

Countries differed in EFW (Fig 3). Using country as a covariate in a quantile regression model, including interaction terms with gestational age, showed significance at the 5% level for all percentiles 5th, 10th, 25th, 50th, 75th, 90th, and 95th (S2 and S3 Tables). This variation due to country was adjusted for maternal characteristics (mother’s age, parity, height, and weight, or with BMI substituting the latter two) and sex of the fetus. To assess the relative contribution of these variables to the variation in EFW, the Wald chi-square statistics in S2 and S3 Tables are informative, e.g., for the 5th percentile (quantile 0.05, first table in S2 Table), as expected, most of the variation (Wald chi-square = 1,797, 1 df) is due to gestational age (linear) as the fetus grows, and there is significant curvature (Wald chi-square = 207, 1 df). Country variation gives Wald chi-square = 36 (9 df); sex of the fetus, 29 (1 df); mother’s height, 26 (1 df); and mother’s age, 22 (1 df), while the Wald chi-square value for weight is negligible. In the same table, the level of significance is listed for these variables, e.g., *p* < 0.001 for country, highly significant. It is clear that variation due to country also occurs independently of maternal characteristics and the sex of the fetus. Fig 3 offers a visualization of country variation for the 10th, 50th, and 90th percentiles for EFW. Country variation in the other ultrasound parameters for the 10th, 50th, and 90th percentiles is presented in S2–S6 Figs. Country differences in EFW percentiles and overall EFW percentiles are presented in S4 Table.

**Fig 4:**
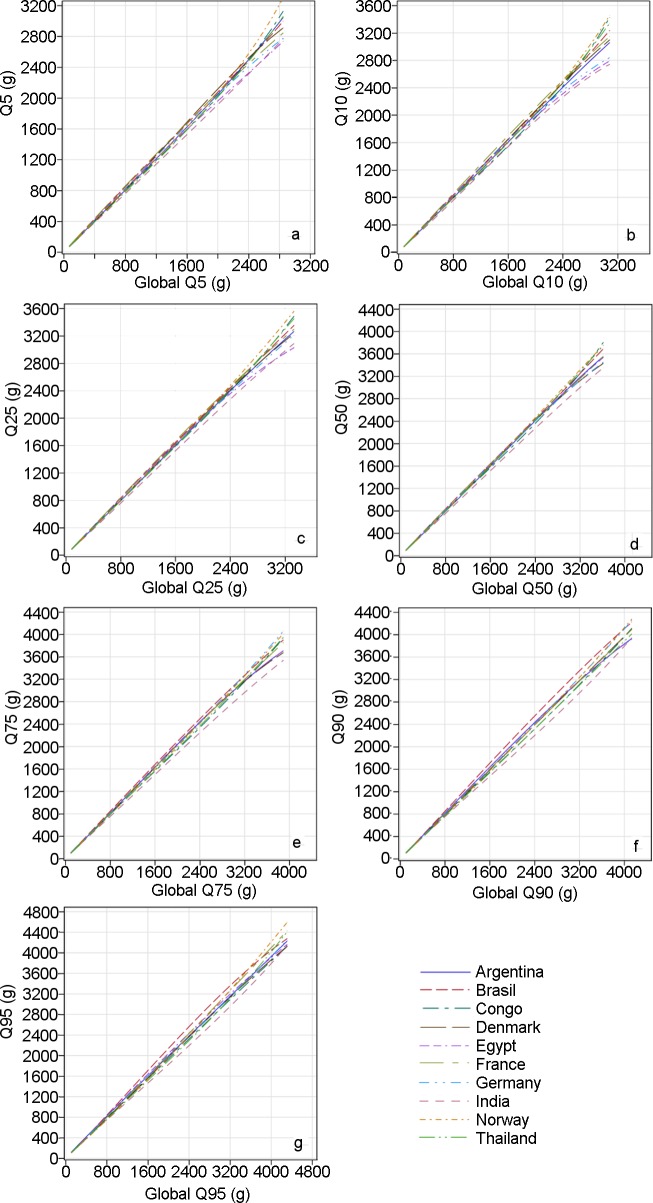
Quantile–quantile plots comparing countries’ distributions with the global distribution of estimated fetal weight. The 5th, 10th, 25th, 50th, 75th, 90th, and 95th percentiles (Q05, Q10, Q25, Q50, Q75, and Q90, respectively) for the distribution of each country are plotted versus the same percentiles of the global distribution (global Q05, global Q10, global Q25, global Q50, global Q75, global Q90, respectively). Congo, Democratic Republic of the Congo.

**Fig 5:**
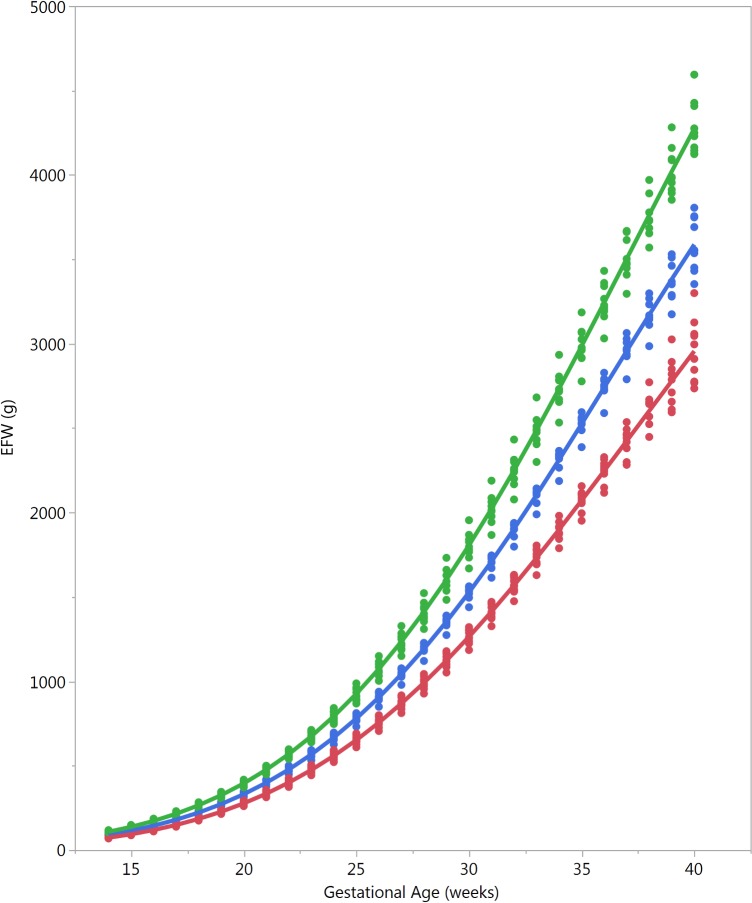
Country differences in estimated fetal weight. Selected percentiles for estimated fetal weight (EFW) for the ten participating countries, showing the magnitude of differences (red, 5th percentile; blue, 50th percentile; green, 95th percentile; each dot denotes a country).

The clinical relevance of the differences between the country quantiles and the global quantiles can be assessed in quantile–quantile plots (Fig 4). These plots are intended to enable the reader to derive the magnitude of difference in grams for any size and country and percentile. For example, consider the quantile–quantile plot for the individual country 0.05 quantile (i.e., the 5th percentile) for EFW versus the global 0.05 quantile: the 5th percentiles at low values of EFW cannot be differentiated because of the relative smallness of EFW at early pregnancy (Fig 4). However, at the end of gestation (high values of EFW), the 5th percentile for Norway is 3,200 g, while the overall 5th percentile is 2,800 g; for France it is 2,800 g, and for Egypt, 2,700 g. Similarly, it can be seen that while the 10th percentile for EFW at the end of gestation for Norway is 3,400 g, it is 2,700 g for India (versus about 3,100 g for the global 10th percentile), showing that a fetus weighing 3,200 g would be below the 10th percentile for Norway but well above it for India. The magnitude of the differences among countries can also be appreciated in Fig 5, where selected country percentiles are shown with the corresponding global percentile curve.

#### Maternal age and maternal height

Maternal age and height seem to be associated with a positive effect on EFW, especially in the lower tail of the distribution, significant at the 5% level, of the order of 2% to 3% for each additional 10 y of age of the mother and 1% to 2% for each additional 10 cm of height (S1D and S1F Fig, without adjusting for country differences).

#### Maternal weight

Maternal weight seems to be associated with a small positive effect on EFW, especially in the higher tail of the distribution, significant at the 5% level, of the order of 1% to 1.5% for each additional 10 kg of weight of the mother (S1E Fig, without adjusting for country differences).

#### Parity (0 versus ≥1)

Parous women had heavier fetuses than nulliparous women, with the disparity being much higher in the lower quantiles of the distribution, of the order of 1% to 3%, significant at the 5% level, and subsiding in the upper quantiles (S1C Fig, without adjusting for country differences).

### Influence of Clinical Conditions on Growth Percentiles

Participants for whom clinical conditions occurred during pregnancy and childbirth were retained in the study. We then assessed the effect of excluding them on the parameter estimates of the quantiles. We excluded successively maternal conditions, fetal malformations, and neonatal conditions and assessed the fit for the global EFW percentiles. The parameter estimates obtained were indistinguishable.

In order to illustrate variation of the clinically relevant 10th and 90th percentiles for EFW, we compiled the values (without any formal comparison) for 24, 28, 32, and 36 wk of gestation from the present study, the NICHD Fetal Growth Studies [19], a study from D. R. Congo [30], and another study from Norway [31] (Table 16). Since the other existing multinational study, the Fetal Growth Longitudinal Study of the Intergrowth-21st Project, did not publish EFW but rather AC, which is a major determinant for EFW, we also compiled 10th and 90th percentiles for AC from relevant studies [18,19,30,32–34] (Table 17).

**Table 16:** The 10th and 90th percentile for estimated fetal weight in relation to other relevant reference values.

Reference Chart	Gestational Week				
	20	24	28	32	36
**10th percentile of EFW (g)**					
US, white^¶^	289	583	1,045	1,686	2,432
D. R. Congo^#^	288	576	1,023	1,624	2,310
**WHO**	**286**	**576**	**1,026**	**1,635**	**2,352**
US, black^¶^	286	559	985	1,579	2,264
Norway*	283	610	1,102	1,730	2,411
US, Hispanic^¶^	279	555	987	1,595	2,298
US, Asian^¶^	275	546	978	1,574	2,262
**90th percentile of EFW (g)**					
Norway*	408	833	1,472	2,304	3,230
US, white^¶^	381	771	1,391	2,276	3,368
**WHO**	**380**	**765**	**1,368**	**2,187**	**3,153**
US, Hispanic^¶^	379	755	1,353	2,209	3,245
US, black^¶^	376	742	1,317	2,135	3,115
US, Asian^¶^	373	737	1,318	2,129	3,111
D. R. Congo^#^	345	700	1,277	2,083	3,032

Percentiles from the present multinational study (bold), a recent multiethnic national study in the US [19], a study from D. R. Congo [30], and another study from Norway [31] are listed according to descending values at 20 wk, but are not formally compared or ranked.

^¶^Buck Louis et al. [19].

^#^Landis et al. [30].

*Johnsen et al. [31].

D. R., Congo, Democratic Republic of the Congo; EFW, estimated fetal weight.

**Table 17:** The 10th and 90th percentile for fetal abdominal circumference in relation to relevant reference values.

Reference Chart	Gestational Week				
	20	24	28	32	36
**10th percentile AC (mm)**					
US, white^¶^	141	185	227	268	306
**WHO**	**139**	**184**	**225**	**260**	**294**
Norway*	139	182	223	262	299
US, Asian^¶^	139	182	221	260	295
US, Hispanic^¶^	138	181	221	262	299
Intergrowth-21st Project^§^	138	179	219	257	291
US, black^¶^	137	179	217	267	293
Thailand^#^	135	177	217	254	290
UK^&^	135	175	213	249	283
**90th percentile AC (mm)**					
Norway*	165	213	259	303	346
US, white^¶^	164	212	258	306	353
US, Hispanic^¶^	163	210	255	303	349
**WHO**	**161**	**210**	**256**	**298**	**340**
US, Asian^¶^	161	208	252	299	343
Thailand^#^	159	208	256	301	339
US, black^¶^	159	205	249	295	340
UK^&^	158	204	248	290	330
Intergrowth-21st Project^§^	158	203	248	291	335

Percentiles from the present multinational study (bold), a recent multinational study (Intergrowth-21st Project), a recent multiethnic study in the US, and three studies from Norway, Thailand, and the United Kingdom are listed according to descending values at 20 wk, but are not formally compared or ranked.

^¶^Buck Louis et al. [19].

*Johnsen et al. [33].

^§^Papageorghiou et al. [18].

^#^Sunsaneevithayakul et al. [34].

^&^Chitty et al. [32].

AC, abdominal circumference; D. R., Congo, Democratic Republic of the Congo.

## Discussion

In this paper we present the WHO fetal growth charts for EFW and common ultrasound biometric measurements intended for international use. They reveal a wide range of variation in human fetal growth across different parts of the world. Significant differences in fetal growth between countries are confirmed by differences in birthweight. Furthermore, the study shows that intrauterine growth is influenced by fetal sex and by maternal age, height, weight, and parity, although these influences explain only partially the differences in growth between countries.

The primary motivation for this study, the fetal component of the WHO Multicentre Growth Reference Study [11], was the need for clinical reference intervals applicable internationally, including for areas of the world where perinatal morbidity and mortality are high, hence the multinational design. Driven by the same motivation, we prioritized ultrasound measurements in common clinical use worldwide, the most prominent being EFW (Fig 1; Table 11). The use of estimated weight in grams is simple and intelligible, which enhances clinical management, facilitates communication within the health care system, and is valuable when counselling patients. In addition to the other common measurements in daily use (BPD, HC, AC, and FL) (Fig 1; Tables 6–9), we established reference intervals for the ratios FL/HC and FL/BPD aimed at facilitating the identification and monitoring of disproportionate fetal head development, e.g., hydrocephaly or microcephaly (Fig 1; Tables 12 and 13). The diagnosis in pregnancies complicated by such conditions is often hampered by uncertainty about gestational age since head size (BPD and HC) is also commonly used for the dating of the pregnancy. FL/HC and particularly FL/BPD are less dependent on gestational age after 20 wk of gestation (Fig 1) and may therefore have diagnostic utility.

A strength of the new growth charts provided by the study (Tables 6–15) is that they are based on multinational data, i.e., ten countries, and therefore are more likely to be applicable internationally than previously published reference intervals for EFW based on single countries. A recent sizeable study found significant variation in fetal growth between Asian, black, Hispanic, and white ethnic groups, with Asian fetuses being the smallest and white fetuses the largest, justifying ethnic-specific growth charts [19]. However, that study was confined to the US. Table 16 demonstrates the relation between studies for the clinically important 10th and 90th percentiles for EFW. The WHO growth chart for all countries lies in the middle of them. Although the present study was not designed to investigate ethnic differences, a limited record of participants’ ethnicity showed a distribution largely according to country (Table 2). Interestingly, there was a significant difference in the growth of EFW between countries that was not explained by maternal factors (Fig 3; S2 Table). While ethnic differences may play a role in this variation, as for the US-based study [19], variation could also be due to differences in diet and cultural and socioeconomic factors commonly associated with particular ethnic groups. These may also have played a role in the US-based study.

Another recently published multinational study by the Intergrowth-21st Project presented biometric growth but not EFW data [18]. We therefore present variation in AC, which is closely linked to EFW and is an important predictor of perinatal outcome [6], for the commonly used cutoffs, the 10th and 90th percentiles (Table 17). Interestingly, the 10th percentile for the Intergrowth-21st Project results seems to fall below that of the WHO study, even though the Intergrowth-21st Project study was carried out according to a strictly “prescriptive” concept to establish so-called optimal fetal growth (low-risk pregnancies with no environmental and nutritional constraints, and excluding all conditions during pregnancy and childbirth that may be associated with effects on fetal growth). The WHO study had a similar recruitment but retained in the analysis pregnancies with maternal, fetal, and neonatal clinical conditions, based on the principle that reference intervals should reflect as closely as possible the population to which they will be applied. Furthermore, we assessed the effect of removing such pregnancies from the dataset and found no identifiable effect on the percentiles. As seen from Table 17, it is as if rigorous selection and exclusions have limited effect, and other uncontrolled factors are responsible for the variation between studies and countries. Apart from random error, systematic error due to differences in ultrasound measurement techniques could influence the differences between the studies. However, these studies had well-trained ultrasound operators specifically instructed for the research procedure using internationally accepted techniques, and this should minimize such error.

Another strength of the present WHO study is the use of quantile regression to establish the reference intervals. Quantile regression makes an inference about regression coefficients for the conditional quantiles of a variable without making assumptions about its distribution: there is no need to assume a particular distribution and to estimate its moments. In consequence, it provides a more direct representation of the observed measurements. This is nicely demonstrated in a recent large study establishing population-specific fetal growth charts [35]. The technique is especially useful when the quantiles vary differently with a covariate such as, in the present study, gestational age. In addition, the method is robust against the effect of outliers and can capture important features of the data that might be missed by models that average across the conditional distribution [25].

Quantile regression is particularly useful in studying distribution changes, and shows in the present study that fetal growth in the population is not symmetrical with gestation. Starting with a higher distribution towards the lower percentiles, EFW shifts to an expanded distribution among the higher percentiles and ends with a noticeable asymmetry near term. The Bowley coefficient for asymmetry changed from −0.016 to +0.111 during that period. We are not sure of the nature of the small negative asymmetry in early pregnancy, but speculate that regulatory functions, such as the process of maternal constraint of fetal growth, change through gestation, i.e., fetuses in the higher percentiles may be exposed to greater influences, which vary with maternal characteristics. This corroborates the differential effects of covariates across the percentiles shown in S1 Fig. We believe that studying distribution dynamics may yield more information on the control of fetal growth.

The study confirmed the biologically interesting facts that fetal sex and maternal height, weight, parity, and age significantly influence fetal growth [31,36,37]. Together with the country differences, the ethnic differences shown in the US population [19], and, not least, the substantial variation in birthweight among carefully selected low-risk pregnancies, these findings document a diversity and plasticity in human prenatal growth dynamics that is only partially understood. There is increasing evidence linking fetal development, and proxies of development such as birthweight, to postnatal health and life course risk of disease [7,9]. This issue is prioritized by the UN and WHO at a time when noncommunicable diseases are becoming global epidemics [10,38]. For example, in our study, birthweights in India were significantly lower than in the other countries, and Indian participants also had the lowest fetal growth and were the shortest mothers. It is known that body composition in Indian newborns contains relatively more fat [39], a pattern that passes across generations [40] and that is linked to increased risk of subsequent type 2 diabetes [41]. It seems clear that the understanding of “optimal” fetal growth needs to incorporate more than birthweight.

To have a single fetal growth chart that fits all pregnancies across the world would require that all fetuses had the same genetic background for growth, that this genetic background was reliably expressed in the mother, and that influences such as nutrition, physical activity, stress, toxicants, and other environmental conditions had similar effects on the genotype in all embryos and fetuses. This is very unlikely: recent research has revealed a range of interactions between the developmental environment and genetic and epigenetic processes [9]. Even influences on fetal growth classically thought to be primarily genetic, such as maternal and paternal height, are complicated by environmental factors. Altitude, climate, geography, other environmental conditions, and the challenges of daily life and nutrition vary around the world. Humans adapt across generations to local conditions, and fetal development adds an important adaptive refinement for the next generation. Secular changes in birthweight and child growth patterns have been shown to accompany social changes [42,43]. Fetal growth charts may thus need to be adjusted to fit the diversity of individuals and populations if they are to be of the greatest clinical utility.

While including ten countries in the present WHO study was a strength compared to previous studies, it still has limitations. The ten population samples, including two in South-East Asia and two in Africa, were included to increase generalizability, but they are still a very limited sample of the global human population. Africa alone has a greater genetic diversity than has the rest of the world [44], and anthropometric variation on that continent is substantial. The present study showed population differences within the pooled dataset, and so the extent to which the results can be extrapolated to other populations, which possibly have other growth dynamics, is at present unknown.

A limitation of the study is that ultrasound measurements were accompanied by a corresponding gestational age exposed on the screen, which could have led to undue changes in the management of the pregnancy and pregnancy duration. However, it was common practice among the sonographers and midwives doing the examination not to pay attention to this gestational age because the department was using other reference values than the one on the screen. On the other hand, part of the ethical commitment of the study was actually to let the mother be informed of any abnormality or deviation of importance discovered, so that it could be taken into account for the management of the pregnancy, and to refer the case to the managing clinician. However, the reported referrals were few and were found not to influence the statistics.

Pooling data is not ideal in the presence of variation among populations, and a single overall growth chart will only partially reflect the individual populations included. Figs 4 and 5 show the variation of country-specific percentiles compared with the corresponding overall percentiles of the study and provide an opportunity to assess the magnitude and clinical relevance of the observed variation. Tables 16 and 17 illustrate a similar pattern when compiling the 10th and 90th percentiles for EFW and AC from various relevant high-quality studies available for clinical use. Although no formal statistical comparison was undertaken, the results of these studies illustrate the distribution that can be found around the world. This gives an impression of a wider spread for the 90th percentile than for the 10th. A similar pattern is found within the WHO study itself: a more obvious diversity between the countries for the 90th percentile than for the 10th percentile (Fig 3). As seen from these figures, variation between countries may increase to several hundred grams towards the end of pregnancy, and may cause misclassifications when the overall percentile is used. Secondly, it seems that population variation in growth is more reflected in the 90th percentile than in the lowest percentiles. Thus, it is possible that the 10th, 5th, and 2.5th percentiles of a pooled study are more universally applicable, while the upper percentiles—90th, 95th, and 97.5th—vary more according to population characteristics and accordingly will be more in need of adjustment, i.e., customization, for use at the population level [37].

It follows that whenever the WHO growth charts, or any reference intervals, are applied to a population, their performance should be checked or tested in order to ensure appropriate use. It is possible to adjust them by changing cutoffs (e.g., from 10th to 5th percentile) to fit clinical needs better, and it is possible to customize the percentiles to country, maternal characteristics, and fetal sex to improve diagnostic performance [45]. A further refinement would be to introduce conditioning terms when using repeated ultrasound measurements for monitoring growth [46,47], i.e., narrowing the expected reference interval for an assessment by conditioning it using a previous measurement. WHO is working on these methods to make them generally available with the growth chart.

If such adjustments and refinements do not suffice to make the growth charts fit clinical needs appropriately, then it may be necessary to establish new high-quality reference intervals for a population. For example, the WHO growth charts and many others are based on populations living at altitudes < 1,500 m. However, millions of people live at higher altitudes, and their physiological adaptations include pregnancy and fetal development. It might be that specific charts will be needed for such populations.

The concept of a “standard,” whether international or national, is often used for instruments and methods to make procedures uniform and to reduce random and systematic error, rather than to set a standard for a biological parameter such as height or bodyweight for the population globally. We are inclined to the view that, while the methodology to define reference ranges or charts for fetal growth needs to be standardized, fetal growth itself is a biological parameter expected to reflect adaptive processes and to change with development, time, location, and environmental conditions. Variation in fetal growth within and between populations should therefore not be ignored.

To apply any growth chart sensibly requires insight, critical attitude, and pragmatism. We believe that the present WHO fetal growth charts can be used internationally, particularly where no local data exist. However, once they are in use, it will be prudent to test the performance of the charts in a particular setting in case adjustments, customization, or replacement with population-specific high-quality reference intervals is needed. With the currently varying degrees of resources, health, and needs around the world, health care professionals have the responsibility of fitting and refining the use of the fetal growth charts to best serve the population in their care.

## Supporting Information

S1 Fig Influence of covariates on estimated fetal weight quantiles. (A) Intercept; (B) fetal sex; (C) parity; (D) maternal age; (E) maternal weight; (F) maternal height; (G) gestational age linear component; (H) gestational age quadratic component; (I) gestational age cubic component. Output of quantile profilers from quantile multivariate regression in the logarithmic scale, presented as the effect of covariates with 95% confidence bands. For binary variables (sex of the fetus and parity), the relative change is between the two categories; for continuous variables, the relative change refers to the increment in EFW resulting from a unit increment of the independent variable (year for maternal age, kilogram for maternal weight, and centimeter for maternal height). Gestational age was included in the model with polynomial terms (linear, quadratic, and cubic).
(DOCX)

S2 Fig Influence of country on fetal growth expressed as the ultrasound measure biparietal diameter. Graphs of the 10th, 50th, and 90th percentiles for the ultrasound measure BPD in millimeters for the ten participating countries.
(TIF)

S3 Fig Influence of country on fetal growth expressed as the ultrasound measure head circumference. Graphs of the 10th, 50th, and 90th percentiles for the ultrasound measure HC in millimeters for the ten participating countries.
(TIF)

S4 Fig Influence of country on fetal growth expressed as the ultrasound measure abdominal circumference. Graphs of the 10th, 50th, and 90th percentiles for the ultrasound measure AC in millimeters for the ten participating countries.
(TIF)

S5 Fig Influence of country on fetal growth expressed as the ultrasound measure femur length. Graphs of the 10th, 50th, and 90th percentiles for the ultrasound measure FL in millimeters for the ten participating countries.
(TIF)

S6 Fig Influence of country on fetal growth expressed as the ultrasound measure humerus length. Graphs of the 10th, 50th, and 90th percentiles for the ultrasound measure HL in millimeters for the ten participating countries.
(TIF)

S1 File Growth charts for the fetal ultrasound measurements biparietal diameter, head circumference, abdominal circumference, femur length, and humerus length; for estimated fetal weight; and for the ratios femur length/head circumference and femur length/biparietal diameter in one Excel file. (XLSX)

S1 Table Compliance of ultrasound visits with protocol, measured by observed versus expected. (DOCX)

S2 Table Variation of estimated fetal weight quantiles due to country, maternal characteristics (age, height, weight, and parity), and sex of the fetus. Output from quantile multivariate regression showing Wald chi-square tests for gestational age; country; the interaction of gestational age and country; sex of the fetus; and maternal characteristics.
(DOCX)

S3 Table Variation of estimated fetal weight quantiles due to country, maternal characteristics (age, BMI, and parity), and sex of the fetus. Output from quantile multivariate regression showing Wald chi-square tests for gestational age; country; the interaction of gestational age and country; sex of the fetus; and maternal characteristics.
(DOCX)

S4 Table Comparison of country percentiles with overall percentiles. The 10th, 50th, and 90th percentiles for overall EFW, and the 95% confidence intervals for the difference between each country’s percentiles and the overall percentiles at 20, 24, 28, 32, and 36 wk of gestational age. The results should be interpreted with caution (the study was not powered for this analysis; multiplicity of inferences implies that the confidence is much lower than 95%).
(DOCX)

## Abbreviations

AC: abdominal circumference
BMI: body mass index
BPD: biparietal diameter
D. R. Congo: Democratic Republic of the Congo
EFW: estimated fetal weight
FL: femur length
HC: head circumference
HL: humerus length
IQR: interquartile range
LMP: last menstrual period
TI: thermal index
