# Correction: The World Health Organization Fetal Growth Charts: A Multinational Longitudinal Study of Ultrasound Biometric Measurements and Estimated Fetal Weight

**DOI:** 10.1371/journal.pmed.1002284

**Published:** 2017-03-24

**Authors:** Torvid Kiserud, Gilda Piaggio, Guillermo Carroli, Mariana Widmer, José Carvalho, Lisa Neerup Jensen, Daniel Giordano, José Guilherme Cecatti, Hany Abdel Aleem, Sameera A. Talegawkar, Alexandra Benachi, Anke Diemert, Antoinette Tshefu Kitoto, Jadsada Thinkhamrop, Pisake Lumbiganon, Ann Tabor, Alka Kriplani, Rogelio Gonzalez, Kurt Hecher, Mark A. Hanson, A. Metin Gülmezoglu, Lawrence D. Platt

The authors discovered a computational error that led to errors in Fig 1, Table 12 and Table 13. The authors have provided corrected versions here.

**Fig 1:**
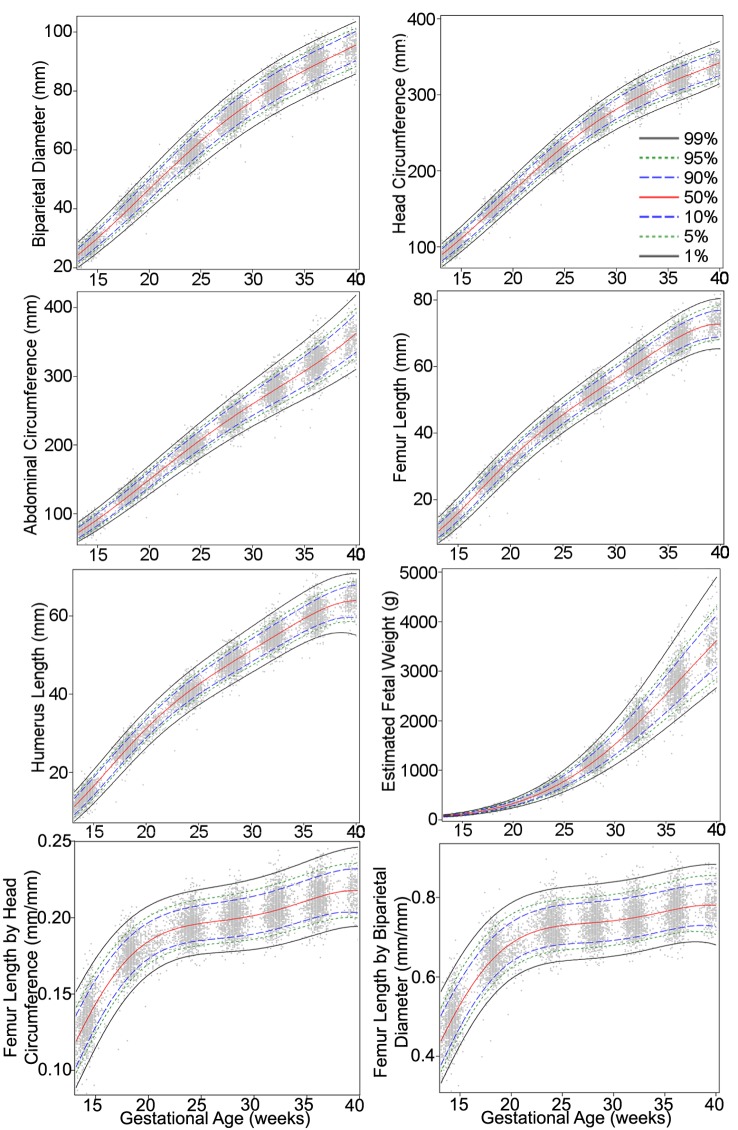
Percentiles for biparietal (outer–inner) diameter, head circumference, abdominal circumference, femur length, humerus length, estimated fetal weight, femur length/head circumference ratio, and femur length/biparietal diameter ratio during gestational weeks 14–40. The percentiles (percent) 1st, 5th, 10th, 50th, 90th, 95th, and 99th (smoothed lines) are based on quantile regression and are shown with the observed values (grey dots).

**Table 12:** Growth chart for fetal femur length/head circumference ratio.

Gestational Age (Weeks)	Femur Length/Biparietal Diameter Ratio by Percentile								
	2.5	5	10	25	50	75	90	95	97.5
**14**	0.39	0.41	0.42	0.45	0.48	0.51	0.54	0.57	0.58
**15**	0.44	0.46	0.47	0.50	0.53	0.56	0.59	0.61	0.63
**16**	0.48	0.50	0.52	0.54	0.57	0.60	0.63	0.65	0.67
**17**	0.52	0.54	0.56	0.58	0.61	0.64	0.66	0.69	0.70
**18**	0.56	0.57	0.59	0.61	0.64	0.67	0.69	0.72	0.73
**19**	0.59	0.60	0.62	0.64	0.66	0.69	0.72	0.74	0.75
**20**	0.61	0.62	0.64	0.66	0.68	0.71	0.74	0.76	0.77
**21**	0.62	0.64	0.65	0.67	0.70	0.73	0.75	0.77	0.78
**22**	0.64	0.65	0.66	0.69	0.71	0.74	0.76	0.78	0.79
**23**	0.65	0.66	0.67	0.69	0.72	0.75	0.77	0.79	0.80
**24**	0.65	0.66	0.68	0.70	0.72	0.75	0.78	0.79	0.80
**25**	0.66	0.67	0.68	0.70	0.73	0.76	0.78	0.79	0.81
**26**	0.66	0.67	0.68	0.71	0.73	0.76	0.78	0.80	0.81
**27**	0.66	0.67	0.69	0.71	0.73	0.76	0.79	0.80	0.81
**28**	0.66	0.67	0.69	0.71	0.74	0.76	0.79	0.80	0.81
**29**	0.66	0.68	0.69	0.71	0.74	0.76	0.79	0.80	0.82
**30**	0.67	0.68	0.69	0.71	0.74	0.77	0.79	0.81	0.82
**31**	0.67	0.68	0.70	0.72	0.74	0.77	0.80	0.81	0.83
**32**	0.68	0.69	0.70	0.72	0.75	0.78	0.80	0.82	0.83
**33**	0.68	0.69	0.70	0.73	0.75	0.78	0.81	0.82	0.84
**34**	0.69	0.70	0.71	0.73	0.76	0.79	0.81	0.83	0.84
**35**	0.69	0.70	0.72	0.74	0.76	0.79	0.82	0.84	0.85
**36**	0.70	0.71	0.72	0.74	0.77	0.80	0.82	0.84	0.86
**37**	0.70	0.71	0.73	0.75	0.77	0.80	0.83	0.85	0.86
**38**	0.70	0.71	0.73	0.75	0.78	0.81	0.83	0.85	0.87
**39**	0.70	0.71	0.73	0.75	0.78	0.81	0.83	0.85	0.87
**40**	0.70	0.71	0.73	0.75	0.78	0.81	0.83	0.85	0.87

**Table 13:** Growth chart for fetal femur length/biparietal diameter.

Gestational Age (Weeks)	Femur Length/Head Circumference Ratio by Percentile								
	2.5	5	10	25	50	75	90	95	97.5
**14**	0.11	0.11	0.11	0.12	0.13	0.14	0.15	0.15	0.16
**15**	0.12	0.12	0.13	0.14	0.14	0.15	0.16	0.16	0.17
**16**	0.13	0.14	0.14	0.15	0.15	0.16	0.17	0.17	0.18
**17**	0.14	0.15	0.15	0.16	0.16	0.17	0.18	0.18	0.19
**18**	0.15	0.16	0.16	0.17	0.17	0.18	0.19	0.19	0.19
**19**	0.16	0.16	0.17	0.17	0.18	0.19	0.19	0.20	0.20
**20**	0.17	0.17	0.17	0.18	0.18	0.19	0.20	0.20	0.20
**21**	0.17	0.17	0.18	0.18	0.19	0.19	0.20	0.20	0.21
**22**	0.17	0.18	0.18	0.19	0.19	0.20	0.20	0.21	0.21
**23**	0.18	0.18	0.18	0.19	0.19	0.20	0.21	0.21	0.21
**24**	0.18	0.18	0.18	0.19	0.19	0.20	0.21	0.21	0.21
**25**	0.18	0.18	0.19	0.19	0.20	0.20	0.21	0.21	0.21
**26**	0.18	0.18	0.19	0.19	0.20	0.20	0.21	0.21	0.22
**27**	0.18	0.18	0.19	0.19	0.20	0.20	0.21	0.21	0.22
**28**	0.18	0.18	0.19	0.19	0.20	0.21	0.21	0.22	0.22
**29**	0.18	0.18	0.19	0.19	0.20	0.21	0.21	0.22	0.22
**30**	0.18	0.19	0.19	0.20	0.20	0.21	0.21	0.22	0.22
**31**	0.18	0.19	0.19	0.20	0.20	0.21	0.22	0.22	0.22
**32**	0.19	0.19	0.19	0.20	0.20	0.21	0.22	0.22	0.23
**33**	0.19	0.19	0.19	0.20	0.21	0.21	0.22	0.22	0.23
**34**	0.19	0.19	0.20	0.20	0.21	0.22	0.22	0.23	0.23
**35**	0.19	0.19	0.20	0.20	0.21	0.22	0.22	0.23	0.23
**36**	0.19	0.20	0.20	0.21	0.21	0.22	0.23	0.23	0.23
**37**	0.19	0.20	0.20	0.21	0.22	0.22	0.23	0.23	0.24
**38**	0.20	0.20	0.20	0.21	0.22	0.22	0.23	0.23	0.24
**39**	0.20	0.20	0.20	0.21	0.22	0.22	0.23	0.24	0.24
**40**	0.19	0.20	0.20	0.21	0.22	0.22	0.23	0.24	0.24
