# Correction: Correction: The World Health Organization Fetal Growth Charts: A Multinational Longitudinal Study of Ultrasound Biometric Measurements and Estimated Fetal Weight

**DOI:** 10.1371/journal.pmed.1002301

**Published:** 2017-04-20

**Authors:** The PLOS Medicine Staff

## Notice of republication

This article was republished on March 31, 2017, to correct an erroneous image within the correction of the original article. Please download this article again to view the correct version. The originally published, uncorrected article and the republished, corrected articles are provided here for reference. The publisher apologizes for the error.

## Supporting information

S1 File Originally published, uncorrected article. (PDF)

S2 File Republished, corrected article. (PDF)

## References

[1] Kiserud T, Piaggio G, Carroli G, Widmer M, Carvalho J, Neerup Jensen L, et al (2017) The World Health Organization Fetal Growth Charts: A Multinational Longitudinal Study of Ultrasound Biometric Measurements and Estimated Fetal Weight. PLoS Med 14(1): e1002220 doi: 10.1371/journal.pmed.1002220 28118360 10.1371/journal.pmed.1002220 PMC5261648

[2] Kiserud T, Piaggio G, Carroli G, Widmer M, Carvalho J, Neerup Jensen L, et al (2017) Correction: The World Health Organization Fetal Growth Charts: A Multinational Longitudinal Study of Ultrasound Biometric Measurements and Estimated Fetal Weight. PLoS Med 14(3): e1002284 doi: 10.1371/journal.pmed.1002284 28339475 10.1371/journal.pmed.1002284 PMC5365101

