# Correction: The World Health Organization Fetal Growth Charts: A Multinational Longitudinal Study of Ultrasound Biometric Measurements and Estimated Fetal Weight

**DOI:** 10.1371/journal.pmed.1003526

**Published:** 2021-01-07

**Authors:** Torvid Kiserud, Gilda Piaggio, Guillermo Carroli, Mariana Widmer, José Carvalho, Lisa Neerup Jensen, Daniel Giordano, José Guilherme Cecatti, Hany Abdel Aleem, Sameera A. Talegawkar, Alexandra Benachi, Anke Diemert, Antoinette Tshefu Kitoto, Jadsada Thinkhamrop, Pisake Lumbiganon, Ann Tabor, Alka Kriplani, Rogelio Gonzalez Perez, Kurt Hecher, Mark A. Hanson, A. Metin Gülmezoglu, Lawrence D. Platt

The title and caption of Table 12 does not correspond to the table content. The correct title and caption is: “Table 12. Growth chart for fetal femur length/biparietal diameter ratio.”

The title and caption of Table 12 does not correspond to the table content. The correct title and caption is: “Table 13. Growth chart for fetal femur length/head circumference ratio.”
