# Supplementary material for: The World Health Organization Fetal Growth Charts: A Multinational Longitudinal Study of Ultrasound Biometric Measurements and Estimated Fetal Weight
Source: PLoS Med. 2017 Jan 24;14(1):e1002220. doi: 10.1371/journal.pmed.1002220 (PMC5261648; doi:10.1371/journal.pmed.1002220)
Supplement: S1 Fig — (A) Intercept; (B) fetal sex; (C) parity; (D) maternal age; (E) maternal weight; (F) maternal height; (G) gestational age linear component; (H) gestational age quadratic component; (I) gestational age cubic component. Output of quantile profilers from quantile multivariate regression in the logarithmic scale, presented as the effect of covariates with 95% confidence bands. For binary variables (sex of the fetus and parity), the relative change is between the two categories; for continuous variables, the relative change refers to the increment in EFW resulting from a unit increment of the independent variable (year for maternal age, kilogram for maternal weight, and centimeter for maternal height). Gestational age was included in the model with polynomial terms (linear, quadratic, and cubic). (DOCX) [file pmed.1002220.s001.docx]

**S1 Figure: Effect of covariates on estimated fetal weight (EFW) quantiles (gagewgagew=quadratic term for gestational age; gagewgagewgagew=cubic term for gestational age)**


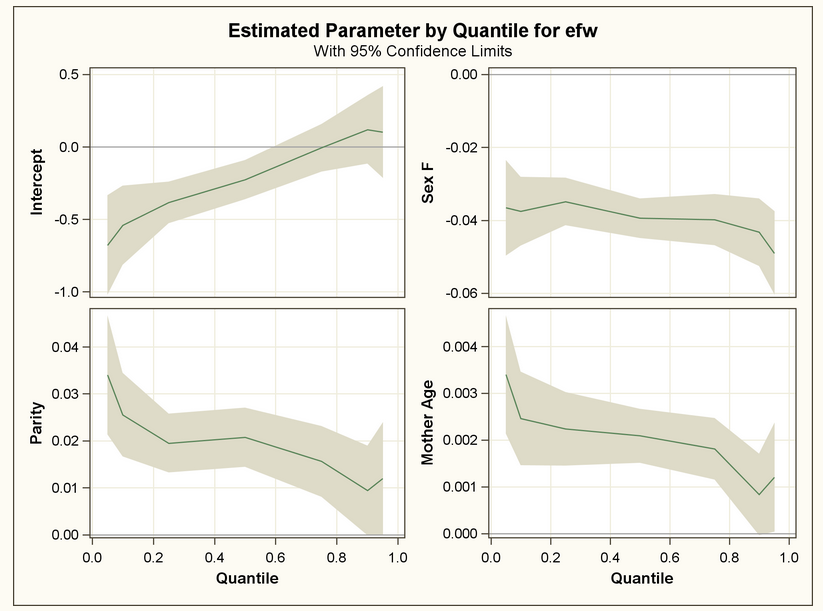


D

C

B

A


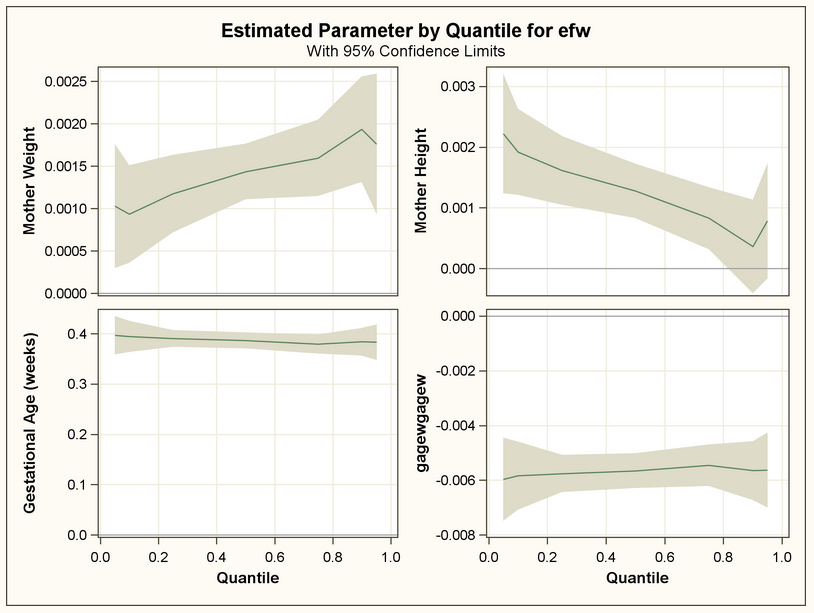


H

G

F

E


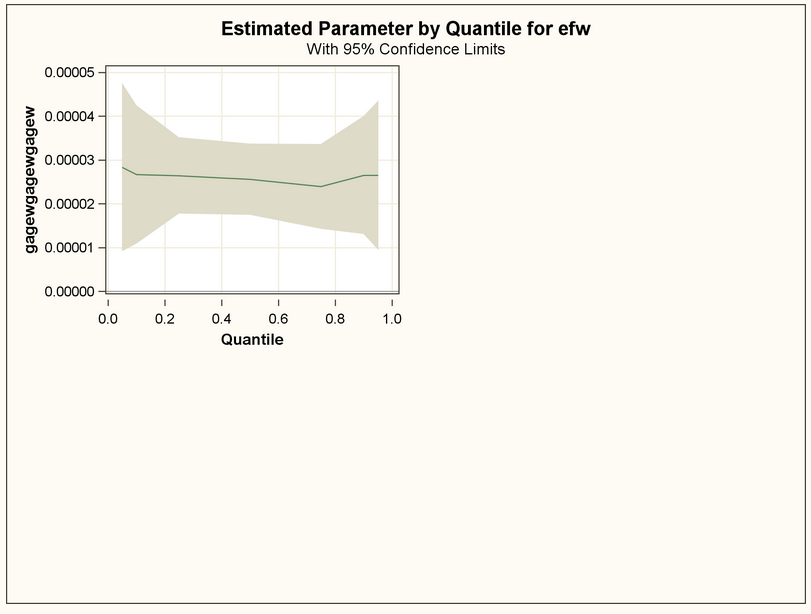


I
