# Supplementary figures and images for: The World Health Organization Fetal Growth Charts: A Multinational Longitudinal Study of Ultrasound Biometric Measurements and Estimated Fetal Weight
Source: PLoS Med. 2017 Jan 24;14(1):e1002220. doi: 10.1371/journal.pmed.1002220 (PMC5261648; doi:10.1371/journal.pmed.1002220)

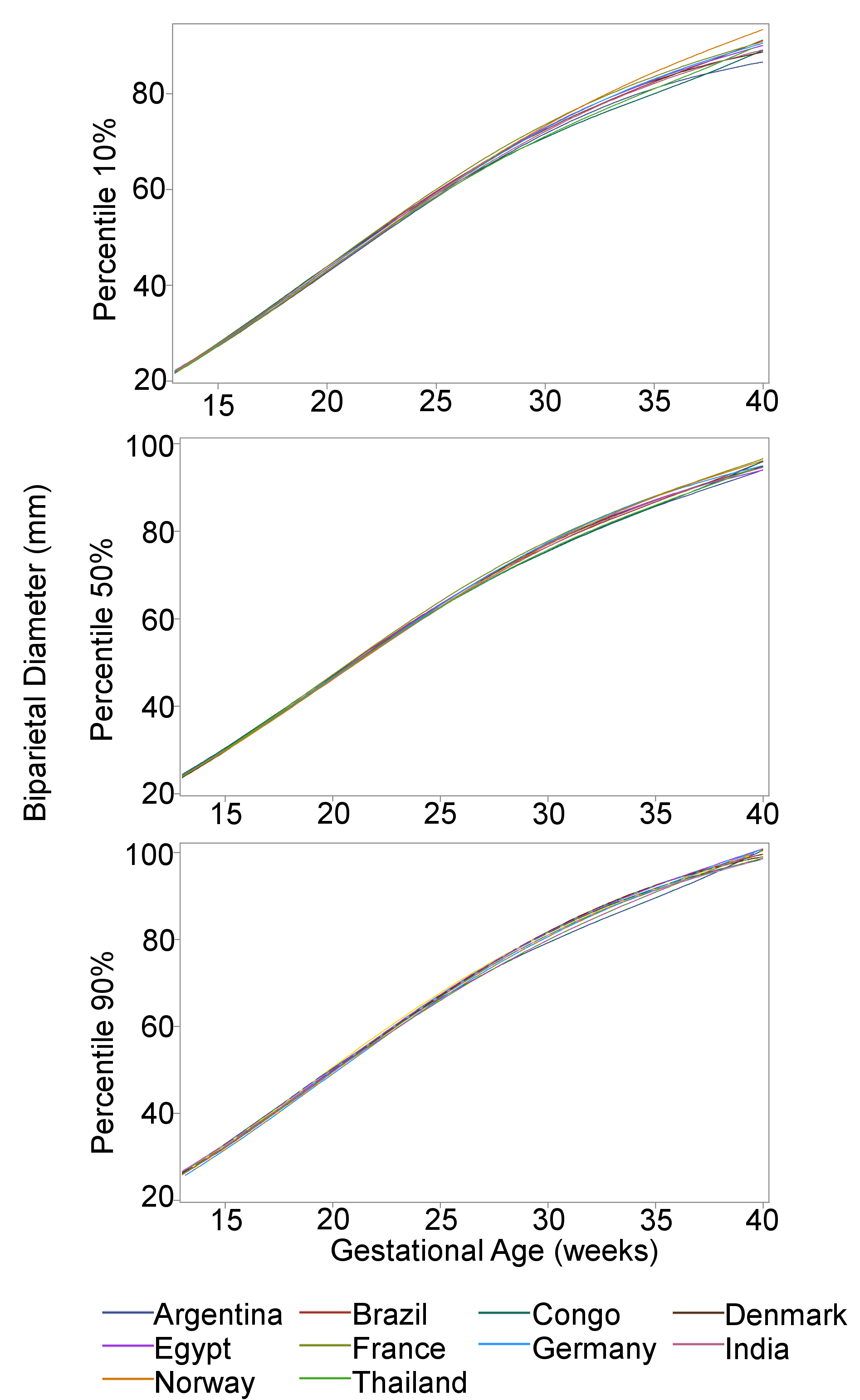

Supplement: S2 Fig — Graphs of the 10th, 50th, and 90th percentiles for the ultrasound measure BPD in millimeters for the ten participating countries. (TIF) [file pmed.1002220.s002.tif]

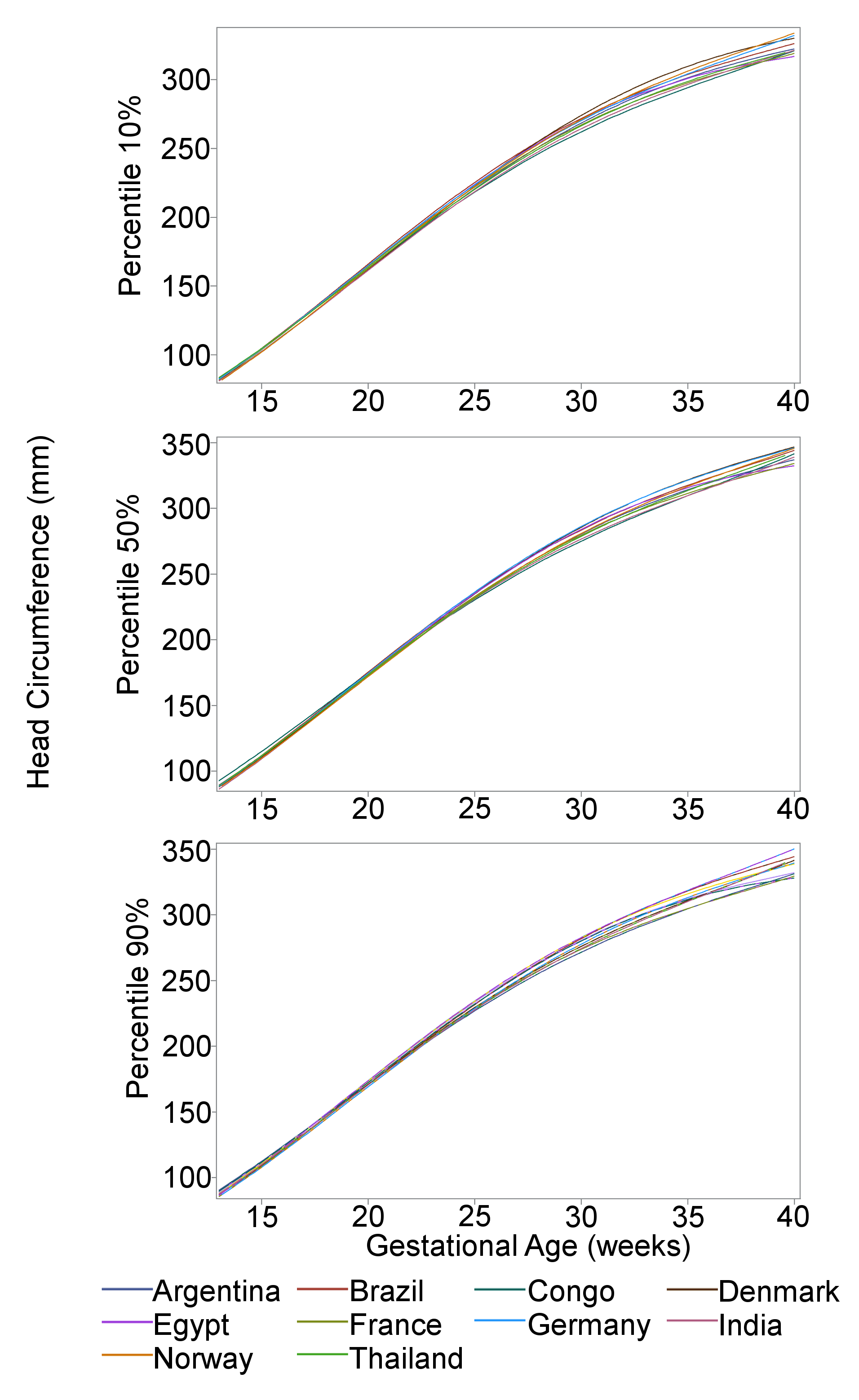

Supplement: S3 Fig — Graphs of the 10th, 50th, and 90th percentiles for the ultrasound measure HC in millimeters for the ten participating countries. (TIF) [file pmed.1002220.s003.tif]

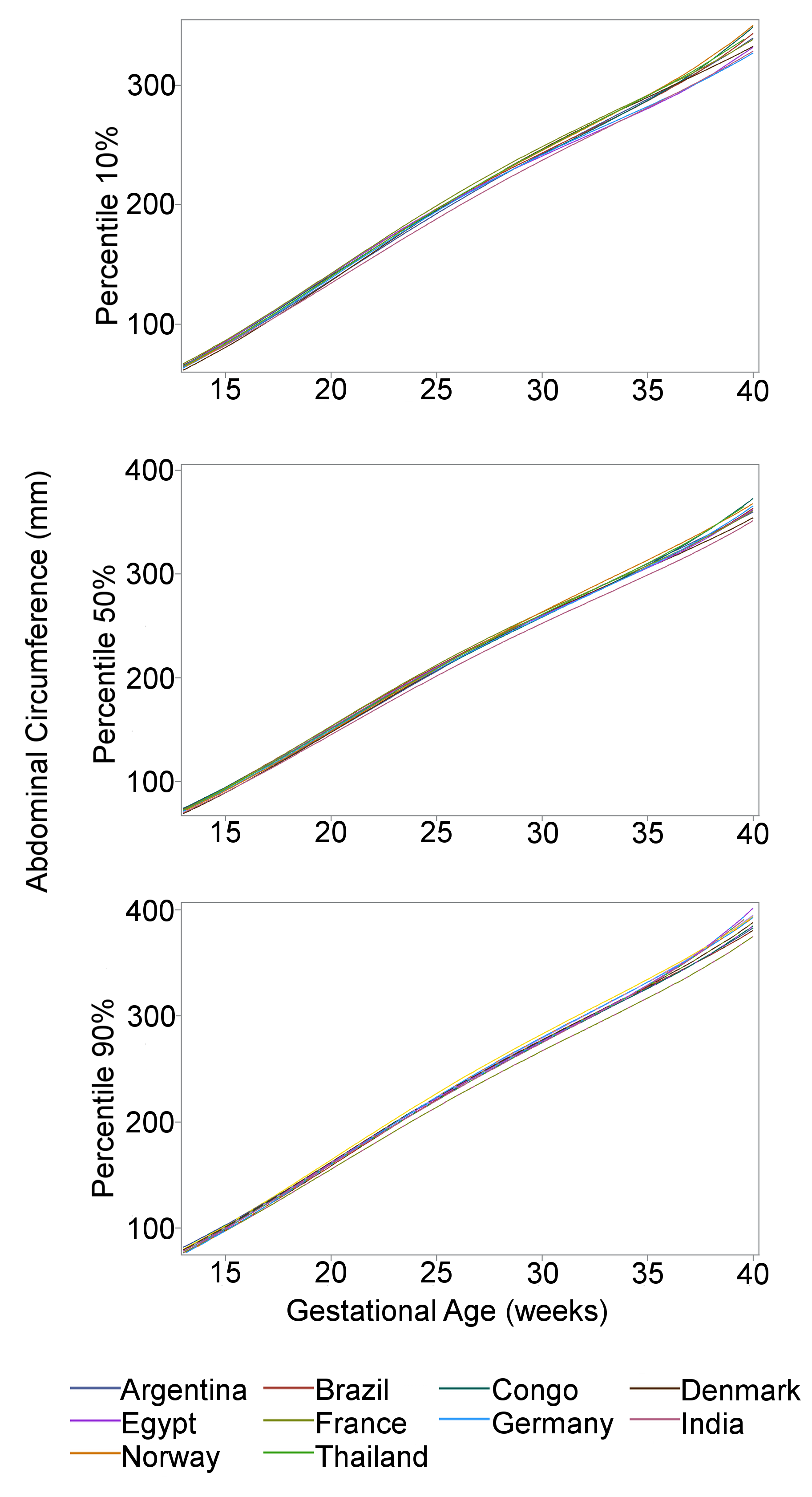

Supplement: S4 Fig — Graphs of the 10th, 50th, and 90th percentiles for the ultrasound measure AC in millimeters for the ten participating countries. (TIF) [file pmed.1002220.s004.tif]

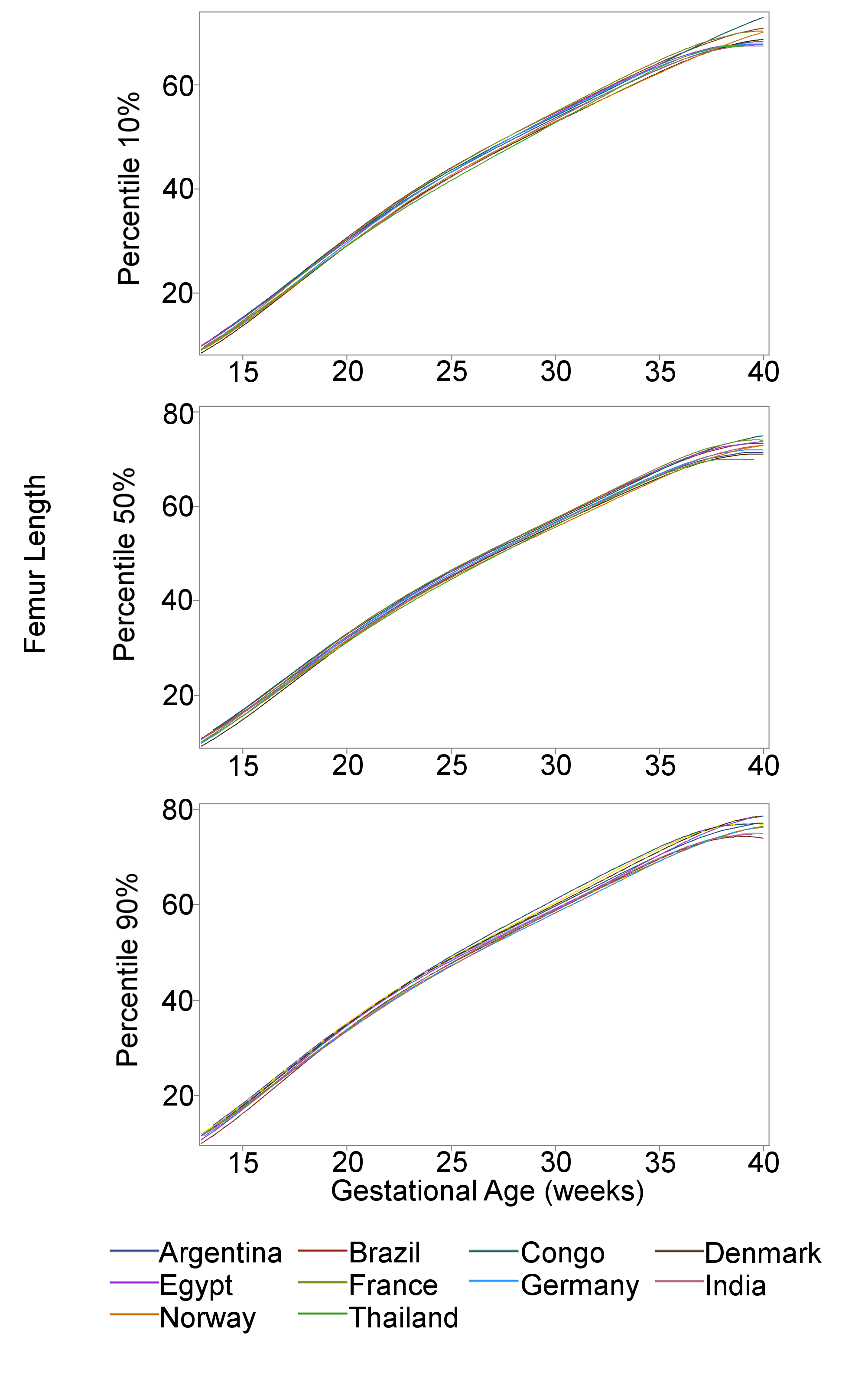

Supplement: S5 Fig — Graphs of the 10th, 50th, and 90th percentiles for the ultrasound measure FL in millimeters for the ten participating countries. (TIF) [file pmed.1002220.s005.tif]

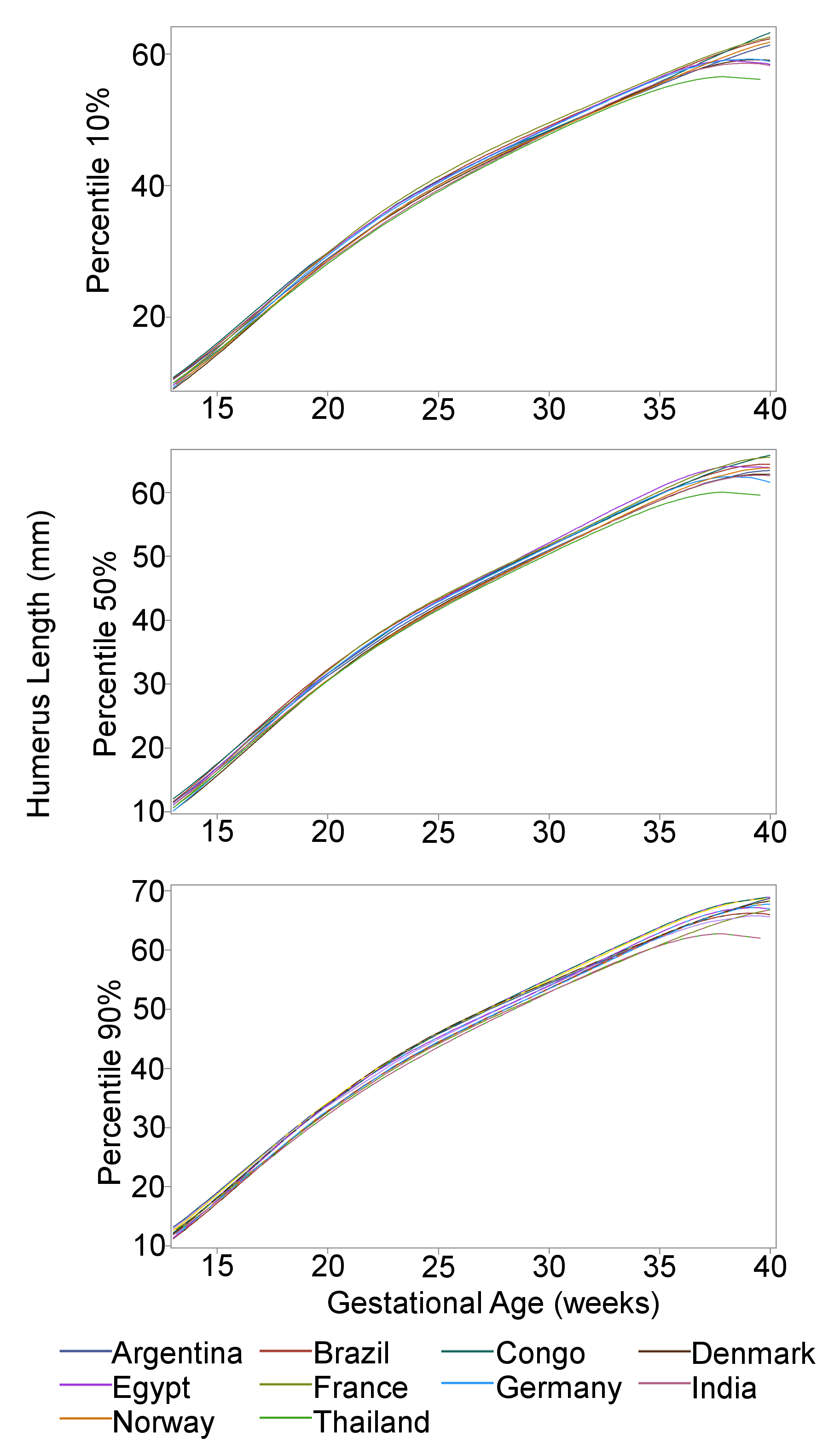

Supplement: S6 Fig — Graphs of the 10th, 50th, and 90th percentiles for the ultrasound measure HL in millimeters for the ten participating countries. (TIF) [file pmed.1002220.s006.tif]
