# Supplementary material for: The World Health Organization Fetal Growth Charts: A Multinational Longitudinal Study of Ultrasound Biometric Measurements and Estimated Fetal Weight
Source: PLoS Med. 2017 Jan 24;14(1):e1002220. doi: 10.1371/journal.pmed.1002220 (PMC5261648; doi:10.1371/journal.pmed.1002220)
Supplement: S1 Table — (DOCX) [file pmed.1002220.s008.docx]

**S1 Table: Compliance of ultrasound visits according to protocol, measured by Observed (Obs) vs. Expected (Exp)**

|  | **US1(14 weeks±1)** | | | **US2(18 weeks±1)** | | | **US3(24 weeks±1)** | | | **US4(28 weeks±1)** | | | **US5(32 weeks±1)** | | | **US6(36 weeks±1)** | | | **US7(40 weeks±1)** | | |
| --- | --- | --- | --- | --- | --- | --- | --- | --- | --- | --- | --- | --- | --- | --- | --- | --- | --- | --- | --- | --- | --- |
| **Country** | **Obs** | **Exp** | **%** | **Obs** | **Exp** | **%** | **Obs** | **Exp** | **%** | **Obs** | **Exp** | **%** | **Obs** | **Exp** | **%** | **Obs** | **Exp** | **%** | **Obs** | **Exp** | **%** |
| Argentina | 142 | 143 | 99.3 | 137 | 141 | 97.2 | 139 | 140 | 99.3 | 138 | 140 | 98.6 | 134 | 137 | 97.8 | 121 | 123 | 98.4 | . | . | . |
| Brazil | 139 | 150 | 92.7 | 141 | 150 | 94 | 92 | 149 | 61.7 | 125 | 143 | 87.4 | 136 | 143 | 95.1 | 117 | 125 | 93.6 | . | . | . |
| Congo | 141 | 150 | 94 | 137 | 146 | 93.8 | 137 | 142 | 96.5 | 133 | 138 | 96.4 | 128 | 136 | 94.1 | 114 | 124 | 91.9 | 3 | 3 | 100 |
| Denmark | 138 | 139 | 99.3 | 58 | 137 | 42.3 | 133 | 137 | 97.1 | 134 | 137 | 97.8 | 130 | 136 | 95.6 | 127 | 129 | 98.4 | 9 | 9 | 100 |
| Egypt | 139 | 160 | 86.9 | 138 | 156 | 88.5 | 134 | 148 | 90.5 | 132 | 145 | 91 | 125 | 139 | 89.9 | 87 | 93 | 93.5 | 1 | 1 | 100 |
| France | 95 | 108 | 88 | 97 | 104 | 93.3 | 87 | 101 | 86.1 | 95 | 101 | 94.1 | 91 | 99 | 91.9 | 80 | 86 | 93 | 1 | 1 | 100 |
| Germany | 135 | 141 | 95.7 | 136 | 141 | 96.5 | 129 | 140 | 92.1 | 139 | 140 | 99.3 | 135 | 140 | 96.4 | 123 | 124 | 99.2 | 1 | 1 | 100 |
| India | 133 | 142 | 93.7 | 136 | 141 | 96.5 | 132 | 140 | 94.3 | 132 | 138 | 95.7 | 112 | 135 | 83 | 78 | 102 | 76.5 | . | . | . |
| Norway | 136 | 137 | 99.3 | 137 | 137 | 100 | 136 | 136 | 100 | 135 | 136 | 99.3 | 136 | 136 | 100 | 127 | 127 | 100 | 15 | 15 | 100 |
| Thailand | 112 | 122 | 91.8 | 105 | 119 | 88.2 | 98 | 116 | 84.5 | 107 | 115 | 93 | 100 | 113 | 88.5 | 89 | 95 | 93.7 | . | . | . |
| **Total** | **1310** | **1392** | **94.1** | **1222** | **1372** | **89.1** | **1217** | **1349** | **90.2** | **1270** | **1333** | **95.3** | **1227** | **1314** | **93.4** | **1063** | **1128** | **94.2** | **30** | **30** | **100** |

Notes:

The median number of ultrasound scans (excluding the screening scans) in all women was 6·0 (range 0–7; mean 5·81 [SD 0·97]).

Compliance in each gestational age window considered by the protocol was between 89,1% and 100%. 72% of women performed all the scans scheduled.

For all biometrical parameters considered in the protocol (except for Transcerebellar Diameter and Foot Length), at least 97.84% of the scans were obtained three or two times from a separately generated ultrasound images.
