# Supplementary material for: The World Health Organization Fetal Growth Charts: A Multinational Longitudinal Study of Ultrasound Biometric Measurements and Estimated Fetal Weight
Source: PLoS Med. 2017 Jan 24;14(1):e1002220. doi: 10.1371/journal.pmed.1002220 (PMC5261648; doi:10.1371/journal.pmed.1002220)
Supplement: S2 Table — Output from quantile multivariate regression showing Wald chi-square tests for gestational age; country; the interaction of gestational age and country; sex of the fetus; and maternal characteristics. (DOCX) [file pmed.1002220.s009.docx]

**S2 Table: Variation of estimated fetal weight (EFW) quantiles due to countries, to maternal characteristics (mother age, mother height, mother weight, parity) and to sex of fetus**

**Model Information**

| Type of model | quantile regression |
| --- | --- |
| Dependent variable | EFW |
| Independent variables (7) | country (10), gestational age (GA, continuous with polynomial terms linear, quadratic and cubic), sex of fetus (F,M), parity (nulliparous, parous), mother age, mother weight, mother height (continuous) |
| Estimation Method | Maximum Likelihood |
| Number of women | 1362 |
| Number of observations | 7299 |

**Quantile 0.05**

**Effect Tests**

| **Source** | **Nparm** | **DF** | **Wald ChiSquare** | **Prob > ChiSquare** |
| --- | --- | --- | --- | --- |
| Country | 9 | 9 | 36,100598 | <,0001* |
| GA | 1 | 1 | 1796,9486 | <,0001* |
| GA*Country | 9 | 9 | 14,460039 | 0,1069 |
| GA*GA | 1 | 1 | 207,80274 | <,0001* |
| GA*GA*Country | 9 | 9 | 11,901438 | 0,2189 |
| GA*GA*GA | 1 | 1 | 0,0626235 | 0,8024 |
| GA*GA*GA*Country | 9 | 9 | 9,5395936 | 0,3890 |
| Mother age | 1 | 1 | 22,355286 | <,0001* |
| Mother height | 1 | 1 | 26,781211 | <,0001* |
| Mother weight | 1 | 1 | 1,6507268 | 0,1989 |
| Sex of fetus | 1 | 1 | 29,104302 | <,0001* |
| Parity | 1 | 1 | 7,8293341 | 0,0051* |

**Quantile 0.10**

**Effect Tests**

| **Source** | **Nparm** | **DF** | **Wald ChiSquare** | **Prob > ChiSquare** |
| --- | --- | --- | --- | --- |
| Country | 9 | 9 | 31,760851 | 0,0002* |
| GA | 1 | 1 | 2561,3171 | <,0001* |
| GA*Country | 9 | 9 | 19,848899 | 0,0189* |
| GA*GA | 1 | 1 | 338,81773 | <,0001* |
| GA*GA*Country | 9 | 9 | 10,731556 | 0,2946 |
| GA*GA*GA | 1 | 1 | 0,3850939 | 0,5349 |
| GA*GA*GA*Country | 9 | 9 | 16,178629 | 0,0632 |
| Mother age | 1 | 1 | 23,021164 | <,0001* |
| Mother height | 1 | 1 | 26,847591 | <,0001* |
| Mother weight | 1 | 1 | 3,5214795 | 0,0606 |
| Sex of fetus | 1 | 1 | 38,298576 | <,0001* |
| Parity | 1 | 1 | 3,5701943 | 0,0588 |

**Quantile 0.25**

**Effect Tests**

| **Source** | **Nparm** | **DF** | **Wald ChiSquare** | **Prob > ChiSquare** |
| --- | --- | --- | --- | --- |
| Country | 9 | 9 | 65,778784 | <,0001* |
| GA | 1 | 1 | 5967,863 | <,0001* |
| GA*Country | 9 | 9 | 26,820009 | 0,0015* |
| GA*GA | 1 | 1 | 789,92795 | <,0001* |
| GA*GA*Country | 9 | 9 | 17,629285 | 0,0397* |
| GA*GA*GA | 1 | 1 | 1,4577573 | 0,2273 |
| GA*GA*GA*Country | 9 | 9 | 22,148738 | 0,0084* |
| Mother age | 1 | 1 | 73,594384 | <,0001* |
| Mother height | 1 | 1 | 62,309118 | <,0001* |
| Mother weight | 1 | 1 | 14,347678 | 0,0002* |
| Sex of fetus | 1 | 1 | 127,57489 | <,0001* |
| Parity | 1 | 1 | 3,8562835 | 0,0496* |

**Quantile 0.50**

**Effect Tests**

| **Source** | **Nparm** | **DF** | **Wald ChiSquare** | **Prob > ChiSquare** |
| --- | --- | --- | --- | --- |
| Country | 9 | 9 | 75,431458 | <,0001* |
| GA | 1 | 1 | 6978,0951 | <,0001* |
| GA*Country | 9 | 9 | 27,689924 | 0,0011* |
| GA*GA | 1 | 1 | 841,77338 | <,0001* |
| GA*GA*Country | 9 | 9 | 33,569029 | 0,0001* |
| GA*GA*GA | 1 | 1 | 1,3421738 | 0,2467 |
| GA*GA*GA*Country | 9 | 9 | 13,426434 | 0,1442 |
| Mother age | 1 | 1 | 68,725892 | <,0001* |
| Mother height | 1 | 1 | 57,161441 | <,0001* |
| Mother weight | 1 | 1 | 35,387179 | <,0001* |
| Sex of fetus | 1 | 1 | 162,17543 | <,0001* |
| Parity | 1 | 1 | 5,1770037 | 0,0229* |

**Quantile 0.75**

**Effect Tests**

| **Source** | **Nparm** | **DF** | **Wald ChiSquare** | **Prob > ChiSquare** |
| --- | --- | --- | --- | --- |
| Country | 9 | 9 | 102,00619 | <,0001* |
| GA | 1 | 1 | 5812,1503 | <,0001* |
| GA*Country | 9 | 9 | 17,45723 | 0,0420* |
| GA*GA | 1 | 1 | 790,05537 | <,0001* |
| GA*GA*Country | 9 | 9 | 26,512752 | 0,0017* |
| GA*GA*GA | 1 | 1 | 1,1982943 | 0,2737 |
| GA*GA*GA*Country | 9 | 9 | 13,350934 | 0,1474 |
| Mother age | 1 | 1 | 42,391141 | <,0001* |
| Mother height | 1 | 1 | 39,471126 | <,0001* |
| Mother weight | 1 | 1 | 21,384577 | <,0001* |
| Sex of fetus | 1 | 1 | 112,97704 | <,0001* |
| Parity | 1 | 1 | 1,0363239 | 0,3087 |

**Quantile 0.90**

**Effect Tests**

| **Source** | **Nparm** | **DF** | **Wald ChiSquare** | **Prob > ChiSquare** |
| --- | --- | --- | --- | --- |
| Country | 9 | 9 | 118,90052 | <,0001* |
| GA | 1 | 1 | 3504,254 | <,0001* |
| GA*Country | 9 | 9 | 17,468963 | 0,0419* |
| GA*GA | 1 | 1 | 476,86534 | <,0001* |
| GA*GA*Country | 9 | 9 | 15,996041 | 0,0670 |
| GA*GA*GA | 1 | 1 | 1,9255797 | 0,1652 |
| GA*GA*GA*Country | 9 | 9 | 8,7255797 | 0,4630 |
| Mother age | 1 | 1 | 28,838406 | <,0001* |
| Mother height | 1 | 1 | 11,098678 | 0,0009* |
| Mother weight | 1 | 1 | 16,435316 | <,0001* |
| Sex of fetus | 1 | 1 | 61,004917 | <,0001* |
| Parity | 1 | 1 | 0,8447156 | 0,3581 |

**Quantile 0.95**

**Effect Tests**

| **Source** | **Nparm** | **DF** | **Wald ChiSquare** | **Prob > ChiSquare** |
| --- | --- | --- | --- | --- |
| Country | 9 | 9 | 87,373108 | <,0001* |
| GA | 1 | 1 | 2897,2671 | <,0001* |
| GA*Country | 9 | 9 | 15,970081 | 0,0675 |
| GA*GA | 1 | 1 | 422,57263 | <,0001* |
| GA*GA*Country | 9 | 9 | 14,309287 | 0,1117 |
| GA*GA*GA | 1 | 1 | 2,3386182 | 0,1262 |
| GA*GA*GA*Country | 9 | 9 | 6,6405724 | 0,6745 |
| Mother age | 1 | 1 | 14,89228 | 0,0001* |
| Mother height | 1 | 1 | 15,316485 | <,0001* |
| Mother weight | 1 | 1 | 33,068222 | <,0001* |
| Sex of fetus | 1 | 1 | 85,877533 | <,0001* |
| Parity | 1 | 1 | 0,0239112 | 0,8771 |
