# Supplementary material for: The World Health Organization Fetal Growth Charts: A Multinational Longitudinal Study of Ultrasound Biometric Measurements and Estimated Fetal Weight
Source: PLoS Med. 2017 Jan 24;14(1):e1002220. doi: 10.1371/journal.pmed.1002220 (PMC5261648; doi:10.1371/journal.pmed.1002220)
Supplement: S3 Table — Output from quantile multivariate regression showing Wald chi-square tests for gestational age; country; the interaction of gestational age and country; sex of the fetus; and maternal characteristics. (DOCX) [file pmed.1002220.s010.docx]

**S3 Table: Variation of estimated fetal weight (EFW) quantiles due to countries, to maternal characteristics (mother age, mother BMI, parity) and to sex of fetus**

**Model Information**

| Type of model | quantile regression |
| --- | --- |
| Dependent variable | EFW |
| Independent variables (7) | country (10), gestational age (GA, continuous with polynomial terms linear, quadratic and cubic), sex of fetus (F,M), parity (nulliparous, parous), mother age, BMI (continuous) |
| Estimation Method | Maximum Likelihood |
| Number of women | 1362 |
| Number of observations | 7299 |

**Quantile 0.05**

**Effect Tests**

| **Source** | **Nparm** | **DF** | **Wald ChiSquare** | **Prob > ChiSquare** |
| --- | --- | --- | --- | --- |
| Country | 9 | 9 | 43,948483 | <,0001* |
| GA | 1 | 1 | 1803,1207 | <,0001* |
| GA*Country | 9 | 9 | 16,082884 | 0,0652 |
| GA*GA | 1 | 1 | 208,89339 | <,0001* |
| GA*GA*Country | 9 | 9 | 15,960851 | 0,0677 |
| GA*GA*GA | 1 | 1 | 0,1137698 | 0,7359 |
| GA*GA*GA*Country | 9 | 9 | 10,669544 | 0,2990 |
| Mother age | 1 | 1 | 16,726077 | <,0001* |
| BMI | 1 | 1 | 0,5919667 | 0,4417 |
| Sex of fetus | 1 | 1 | 24,492794 | <,0001* |
| Parity | 1 | 1 | 13,961158 | 0,0002* |

**Quantile 0.10**

**Effect Tests**

| **Source** | **Nparm** | **DF** | **Wald ChiSquare** | **Prob > ChiSquare** |
| --- | --- | --- | --- | --- |
| Country | 9 | 9 | 41,111397 | <,0001* |
| GA | 1 | 1 | 2646,0198 | <,0001* |
| GA*Country | 9 | 9 | 16,904563 | 0,0502 |
| GA*GA | 1 | 1 | 354,27739 | <,0001* |
| GA*GA*Country | 9 | 9 | 10,53334 | 0,3091 |
| GA*GA*GA | 1 | 1 | 0,8420906 | 0,3588 |
| GA*GA*GA*Country | 9 | 9 | 15,136977 | 0,0872 |
| Mother age | 1 | 1 | 24,151429 | <,0001* |
| BMI | 1 | 1 | 2,1652476 | 0,1412 |
| Sex of fetus | 1 | 1 | 43,226111 | <,0001* |
| Parity | 1 | 1 | 4,1845607 | 0,0408* |

**Quantile 0.25**

**Effect Tests**

| **Source** | **Nparm** | **DF** | **Wald ChiSquare** | **Prob > ChiSquare** |
| --- | --- | --- | --- | --- |
| Country | 9 | 9 | 79,977847 | <,0001* |
| GA | 1 | 1 | 5512,627 | <,0001* |
| GA*Country | 9 | 9 | 20,597887 | 0,0146* |
| GA*GA | 1 | 1 | 702,17609 | <,0001* |
| GA*GA*Country | 9 | 9 | 17,325948 | 0,0438* |
| GA*GA*GA | 1 | 1 | 0,850827 | 0,3563 |
| GA*GA*GA*Country | 9 | 9 | 15,12067 | 0,0877 |
| Mother age | 1 | 1 | 63,895705 | <,0001* |
| BMI | 1 | 1 | 8,8259946 | 0,0030* |
| Sex of fetus | 1 | 1 | 116,649 | <,0001* |
| Parity | 1 | 1 | 3,7493675 | 0,0528 |

**Quantile 0.50**

**Effect Tests**

| **Source** | **Nparm** | **DF** | **Wald ChiSquare** | **Prob > ChiSquare** |
| --- | --- | --- | --- | --- |
| Country | 9 | 9 | 75,513516 | <,0001* |
| GA | 1 | 1 | 6576,3869 | <,0001* |
| GA*Country | 9 | 9 | 26,14632 | 0,0019* |
| GA*GA | 1 | 1 | 825,84817 | <,0001* |
| GA*GA*Country | 9 | 9 | 24,71086 | 0,0033* |
| GA*GA*GA | 1 | 1 | 0,8420691 | 0,3588 |
| GA*GA*GA*Country | 9 | 9 | 16,263762 | 0,0616 |
| Mother age | 1 | 1 | 70,206299 | <,0001* |
| BMI | 1 | 1 | 26,569306 | <,0001* |
| Sex of fetus | 1 | 1 | 158,19098 | <,0001* |
| Parity | 1 | 1 | 2,7805565 | 0,0954 |

**Quantile 0.75**

**Effect Tests**

| **Source** | **Nparm** | **DF** | **Wald ChiSquare** | **Prob > ChiSquare** |
| --- | --- | --- | --- | --- |
| Country | 9 | 9 | 104,06548 | <,0001* |
| GA | 1 | 1 | 5410,7021 | <,0001* |
| GA*Country | 9 | 9 | 14,252978 | 0,1136 |
| GA*GA | 1 | 1 | 712,11627 | <,0001* |
| GA*GA*Country | 9 | 9 | 21,730389 | 0,0098* |
| GA*GA*GA | 1 | 1 | 1,1422519 | 0,2852 |
| GA*GA*GA*Country | 9 | 9 | 10,748527 | 0,2933 |
| Mother age | 1 | 1 | 46,423839 | <,0001* |
| BMI | 1 | 1 | 15,023835 | 0,0001* |
| Sex of fetus | 1 | 1 | 97,2721 | <,0001* |
| Parity | 1 | 1 | 2,4358882 | 0,1186 |

**Quantile 0.90**

**Effect Tests**

| **Source** | **Nparm** | **DF** | **Wald ChiSquare** | **Prob > ChiSquare** |
| --- | --- | --- | --- | --- |
| Country | 9 | 9 | 126,3553 | <,0001* |
| GA | 1 | 1 | 3778,833 | <,0001* |
| GA*Country | 9 | 9 | 13,648714 | 0,1354 |
| GA*GA | 1 | 1 | 488,91287 | <,0001* |
| GA*GA*Country | 9 | 9 | 21,77151 | 0,0096* |
| GA*GA*GA | 1 | 1 | 2,287161 | 0,1304 |
| GA*GA*GA*Country | 9 | 9 | 6,1264167 | 0,7272 |
| Mother age | 1 | 1 | 30,766858 | <,0001* |
| BMI | 1 | 1 | 13,599324 | 0,0002* |
| Sex of fetus | 1 | 1 | 72,084391 | <,0001* |
| Parity | 1 | 1 | 0,9677007 | 0,3253 |

**Quantile 0.95**

**Effect Tests**

| **Source** | **Nparm** | **DF** | **Wald ChiSquare** | **Prob > ChiSquare** |
| --- | --- | --- | --- | --- |
| Country | 9 | 9 | 64,578351 | <,0001* |
| GA | 1 | 1 | 2210,32 | <,0001* |
| GA*Country | 9 | 9 | 15,08442 | 0,0886 |
| GA*GA | 1 | 1 | 316,40053 | <,0001* |
| GA*GA*Country | 9 | 9 | 9,4061277 | 0,4007 |
| GA*GA*GA | 1 | 1 | 1,2708399 | 0,2596 |
| GA*GA*GA*Country | 9 | 9 | 5,2945951 | 0,8079 |
| Mother age | 1 | 1 | 20,363748 | <,0001* |
| BMI | 1 | 1 | 11,097386 | 0,0009* |
| Sex of fetus | 1 | 1 | 49,519101 | <,0001* |
| Parity | 1 | 1 | 1,104112 | 0,2934 |
