# Supplementary material for: The World Health Organization Fetal Growth Charts: A Multinational Longitudinal Study of Ultrasound Biometric Measurements and Estimated Fetal Weight
Source: PLoS Med. 2017 Jan 24;14(1):e1002220. doi: 10.1371/journal.pmed.1002220 (PMC5261648; doi:10.1371/journal.pmed.1002220)
Supplement: S4 Table — The 10th, 50th, and 90th percentiles for overall EFW, and the 95% confidence intervals for the difference between each country’s percentiles and the overall percentiles at 20, 24, 28, 32, and 36 wk of gestational age. The results should be interpreted with caution (the study was not powered for this analysis; multiplicity of inferences implies that the confidence is much lower than 95%). (DOCX) [file pmed.1002220.s011.docx]

| **Country** | **Gestational Age (weeks)** | | | | | | | | | |
| --- | --- | --- | --- | --- | --- | --- | --- | --- | --- | --- |
|  | **20** | | **24** | | **28** | | **32** | | **36** | |
|  | **Lower 95%CI** | **Upper 95%CI** | **Lower 95%CI** | **Upper 95%CI** | **Lower 95%CI** | **Upper 95%CI** | **Lower 95%CI** | **Upper 95%CI** | **Lower 95%CI** | **Upper 95%CI** |
| **Global 10% percentile** | **286** | | **576** | | **1026** | | **1635** | | **2352** | |
| **Difference in 10% percentile of each country versus global 10% percentile** | | | | | | | | | | |
| **Argentina** | -7.6 | -2.0 | -13.1 | -7.5 | -15.5 | -9.9 | -10.6 | -5.0 | -1.0 | 4.6 |
| **Brazil** | 11.7 | 17.3 | 23.1 | 28.7 | 31.3 | 36.8 | 33.8 | 39.4 | 42.9 | 48.5 |
| **Congo** | 6.4 | 12.0 | -2.9 | 2.7 | -32.1 | -26.5 | -58.3 | -52.7 | 5.2 | 10.8 |
| **Denmark** | -9.6 | -4.0 | -5.9 | -0.3 | 12.1 | 17.7 | 43.3 | 48.9 | 61.3 | 66.9 |
| **Egypt** | 4.8 | 10.4 | 14.0 | 19.6 | 21.3 | 26.9 | 3.6 | 9.2 | -84.1 | -78.4 |
| **France** | 14.4 | 20.0 | 33.5 | 39.0 | 62.1 | 67.7 | 92.3 | 97.8 | 102.2 | 107.8 |
| **Germany** | -4.8 | 0.8 | 2.7 | 8.3 | 19.2 | 24.8 | 24.4 | 30.0 | -40.4 | -34.8 |
| **India** | -23.4 | -17.8 | -41.4 | -35.8 | -55.0 | -49.4 | -70.2 | -64.6 | -134.8 | -129.1 |
| **Norway** | -1.0 | 4.6 | -1.3 | 4.3 | -6.7 | -1.2 | 0.8 | 6.4 | 84.0 | 89.6 |
| **Thailand** | -5.3 | 0.3 | -11.6 | -6.0 | -18.9 | -13.3 | -9.4 | -3.8 | 58.8 | 64.4 |

| **Country** | **Gestational Age (weeks)** | | | | | | | | | |
| --- | --- | --- | --- | --- | --- | --- | --- | --- | --- | --- |
|  | **20** | | **24** | | **28** | | **32** | | **36** | |
|  | **Lower 95%CI** | **Upper 95%CI** | **Lower 95%CI** | **Upper 95%CI** | **Lower 95%CI** | **Upper 95%CI** | **Lower 95%CI** | **Upper 95%CI** | **Lower 95%CI** | **Upper 95%CI** |
| **Global 50% percentile** | **330** | | **665** | | **1189** | | **1901** | | **2745** | |
| **Difference in 50% percentile of each country versus global 50% percentile** | | | | | | | | | | |
| **Argentina** | -7.7 | -2.1 | -14.4 | -8.9 | -14.9 | -9.3 | -5.6 | 0.0 | -3.8 | 1.7 |
| **Brazil** | 14.7 | 20.3 | 28.0 | 33.5 | 35.4 | 41.0 | 32.4 | 38.0 | 32.0 | 37.6 |
| **Congo** | 13.0 | 18.6 | 10.6 | 16.2 | -13.4 | -7.9 | -48.0 | -42.4 | -27.4 | -21.9 |
| **Denmark** | -13.1 | -7.6 | -11.1 | -5.5 | 2.6 | 8.1 | 16.1 | 21.7 | -18.1 | -12.5 |
| **Egypt** | 6.7 | 12.3 | 18.0 | 23.6 | 30.7 | 36.3 | 32.7 | 38.2 | 3.8 | 9.3 |
| **France** | 11.4 | 17.0 | 25.9 | 31.5 | 33.1 | 38.7 | 18.7 | 24.3 | -20.7 | -15.1 |
| **Germany** | -4.9 | 0.7 | 0.9 | 6.4 | 12.5 | 18.1 | 17.0 | 22.5 | -22.8 | -17.2 |
| **India** | -20.3 | -14.8 | -42.2 | -36.7 | -71.5 | -65.9 | -107.6 | -102.0 | -160.9 | -155.4 |
| **Norway** | -11.4 | -5.8 | -14.4 | -8.9 | -6.4 | -0.9 | 23.4 | 29.0 | 77.9 | 83.5 |
| **Thailand** | -7.9 | -2.4 | -11.3 | -5.8 | -11.9 | -6.3 | 0.1 | 5.7 | 41.5 | 47.1 |

| **Country** | **Gestational Age (weeks)** | | | | | | | | | |
| --- | --- | --- | --- | --- | --- | --- | --- | --- | --- | --- |
|  | **20** | | **24** | | **28** | | **32** | | **36** | |
|  | **Lower 95%CI** | **Upper 95%CI** | **Lower 95%CI** | **Upper 95%CI** | **Lower 95%CI** | **Upper 95%CI** | **Lower 95%CI** | **Upper 95%CI** | **Lower 95%CI** | **Upper 95%CI** |
| **Global 90% percentile** | **380** | | **765** | | **1368** | | **2187** | | **3153** | |
| **Difference in 90% percentile of each country versus global 90% percentile** | | | | | | | | | | |
| **Argentina** | 7.5 | 13.1 | 16.5 | 22.1 | 29.5 | 35.1 | 31.0 | 36.6 | -21.7 | -16.1 |
| **Brazil** | 15.7 | 21.3 | 44.1 | 49.7 | 88.5 | 94.1 | 135.6 | 141.2 | 153.1 | 158.7 |
| **Congo** | 4.9 | 10.5 | -8.4 | -2.8 | -39.6 | -34.0 | -79.8 | -74.2 | -92.6 | -87.1 |
| **Denmark** | -13.5 | -7.9 | -14.7 | -9.1 | -14.6 | -9.0 | -15.6 | -10.0 | -20.5 | -14.9 |
| **Egypt** | 2.2 | 7.8 | 12.6 | 18.2 | 29.9 | 35.5 | 36.3 | 41.9 | -17.2 | -11.6 |
| **France** | 2.8 | 8.4 | 5.4 | 11.0 | 2.1 | 7.7 | -11.1 | -5.5 | -29.3 | -23.7 |
| **Germany** | -4.3 | 1.3 | -2.5 | 3.1 | -2.7 | 2.9 | -0.3 | 5.3 | 27.0 | 32.6 |
| **India** | -17.2 | -11.6 | -46.4 | -40.8 | -105.2 | -99.6 | -186.0 | -180.4 | -243.1 | -237.5 |
| **Norway** | -17.4 | -11.8 | -24.4 | -18.8 | -28.8 | -23.3 | -19.0 | -13.4 | 29.2 | 34.7 |
| **Thailand** | -16.1 | -10.5 | -33.2 | -27.6 | -57.6 | -52.0 | -84.0 | -78.4 | -104.2 | -98.6 |
