# Supplementary material for: Correction: Correction: The World Health Organization Fetal Growth Charts: A Multinational Longitudinal Study of Ultrasound Biometric Measurements and Estimated Fetal Weight
Source: PLoS Med. 2017 Apr 20;14(4):e1002301. doi: 10.1371/journal.pmed.1002301 (PMC5398478; doi:10.1371/journal.pmed.1002301)
Supplement: S2 File — (PDF) [file pmed.1002301.s002.pdf]

PLOS | MEDICINE

CORRECTION

Correction: The World Health Organization Fetal Growth Charts: A Multinational Longitudinal Study of Ultrasound Biometric Measurements and Estimated Fetal Weight

Torvid Kiserud, Gilda Piaggio, Guillermo Carroli, Mariana Widmer, José Carvalho, Lisa Neerup Jensen, Daniel Giordano, José Guilherme Cecatti, Hany Abdel Aleem, Sameera A. Talegawkar, Alexandra Benachi, Anke Diemert, Antoinette Tshefu Kitoto, Jadsada Thinkhamrop, Pisake Lumbiganon, Ann Tabor, Alka Kriplani, Rogelio Gonzalez, Kurt Hecher, Mark A. Hanson, A. Metin Gülmezoglu, Lawrence D. Platt

The authors discovered a computational error that led to errors in Fig 1, Table 12 and Table 13. The authors have provided corrected versions here.

Check for updates

OPEN ACCESS

Citation: Kiserud T, Piaggio G, Carroli G, Widmer M, Carvalho J, Neerup Jensen L, et al. (2017) Correction: The World Health Organization Fetal Growth Charts: A Multinational Longitudinal Study of Ultrasound Biometric Measurements and Estimated Fetal Weight. PLoS Med 14(3): e1002284. https://doi.org/10.1371/journal.pmed.1002284

Published: March 24, 2017

Copyright: © 2017 Kiserud et al. This is an open access article distributed under the terms of the Creative Commons Attribution License, which permits unrestricted use, distribution, and reproduction in any medium, provided the original author and source are credited.

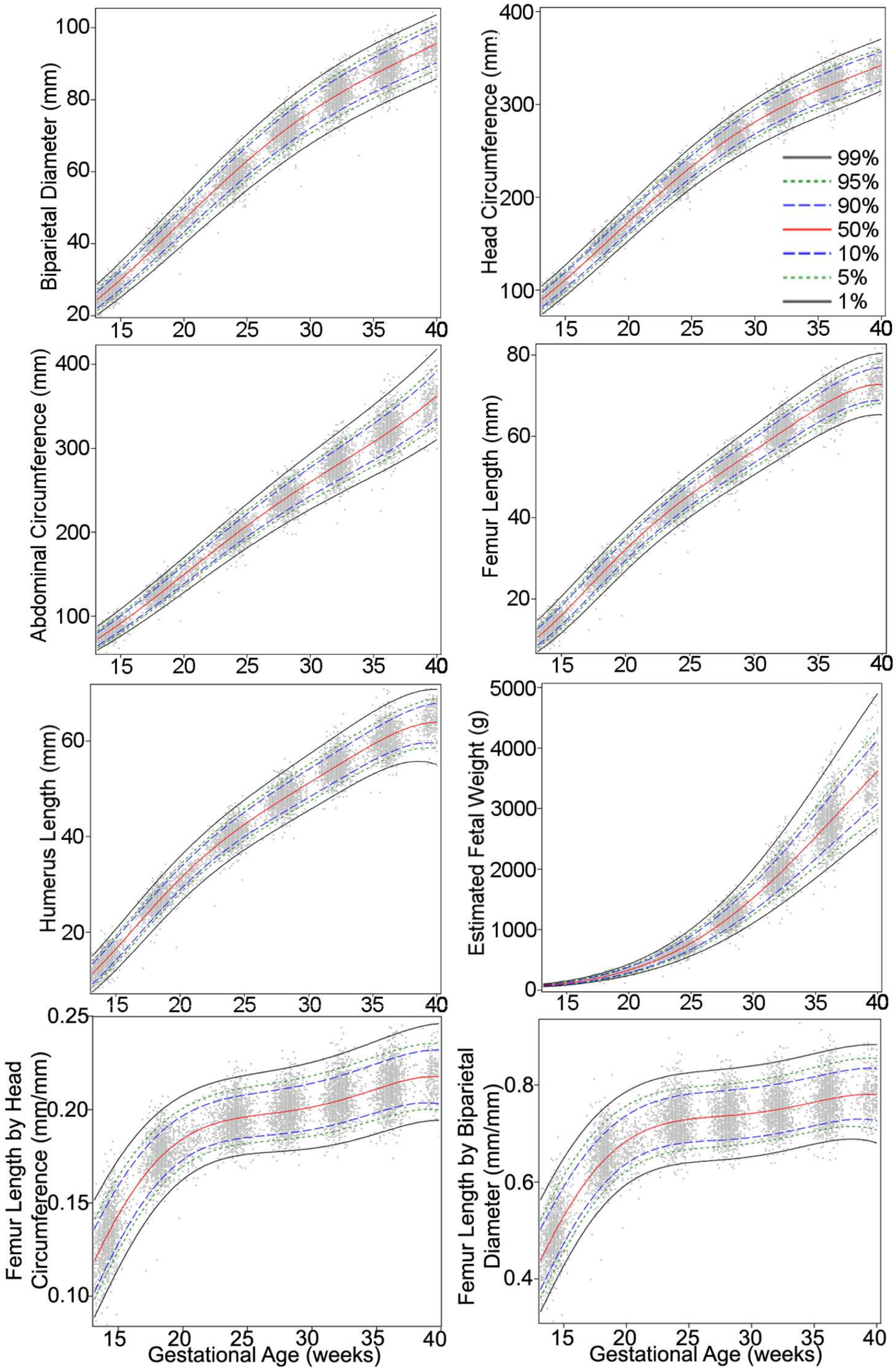

Fig 1. Percentiles for biparietal (outer–inner) diameter, head circumference, abdominal circumference, femur length, humerus length, estimated fetal weight, femur length/head circumference ratio, and femur length/biparietal diameter ratio during gestational weeks 14–40. The percentiles (percent) 1st, 5th, 10th, 50th, 90th, 95th, and 99th (smoothed lines) are based on quantile regression and are shown with the observed values (grey dots).

https://doi.org/10.1371/journal.pmed.1002284.g001

Table 12. Growth chart for fetal femur length/head circumference ratio.

Gestational Age (Weeks)	Femur Length/Biparietal Diameter Ratio by Percentile								
	2.5	5	10	25	50	75	90	95	97.5
14	0.39	0.41	0.42	0.45	0.48	0.51	0.54	0.57	0.58
15	0.44	0.46	0.47	0.50	0.53	0.56	0.59	0.61	0.63
16	0.48	0.50	0.52	0.54	0.57	0.60	0.63	0.65	0.67
17	0.52	0.54	0.56	0.58	0.61	0.64	0.66	0.69	0.70
18	0.56	0.57	0.59	0.61	0.64	0.67	0.69	0.72	0.73
19	0.59	0.60	0.62	0.64	0.66	0.69	0.72	0.74	0.75
20	0.61	0.62	0.64	0.66	0.68	0.71	0.74	0.76	0.77
21	0.62	0.64	0.65	0.67	0.70	0.73	0.75	0.77	0.78
22	0.64	0.65	0.66	0.69	0.71	0.74	0.76	0.78	0.79
23	0.65	0.66	0.67	0.69	0.72	0.75	0.77	0.79	0.80
24	0.65	0.66	0.68	0.70	0.72	0.75	0.78	0.79	0.80
25	0.66	0.67	0.68	0.70	0.73	0.76	0.78	0.79	0.81
26	0.66	0.67	0.68	0.71	0.73	0.76	0.78	0.80	0.81
27	0.66	0.67	0.69	0.71	0.73	0.76	0.79	0.80	0.81
28	0.66	0.67	0.69	0.71	0.74	0.76	0.79	0.80	0.81
29	0.66	0.68	0.69	0.71	0.74	0.76	0.79	0.80	0.82
30	0.67	0.68	0.69	0.71	0.74	0.77	0.79	0.81	0.82
31	0.67	0.68	0.70	0.72	0.74	0.77	0.80	0.81	0.83
32	0.68	0.69	0.70	0.72	0.75	0.78	0.80	0.82	0.83
33	0.68	0.69	0.70	0.73	0.75	0.78	0.81	0.82	0.84
34	0.69	0.70	0.71	0.73	0.76	0.79	0.81	0.83	0.84
35	0.69	0.70	0.72	0.74	0.76	0.79	0.82	0.84	0.85
36	0.70	0.71	0.72	0.74	0.77	0.80	0.82	0.84	0.86
37	0.70	0.71	0.73	0.75	0.77	0.80	0.83	0.85	0.86
38	0.70	0.71	0.73	0.75	0.78	0.81	0.83	0.85	0.87
39	0.70	0.71	0.73	0.75	0.78	0.81	0.83	0.85	0.87
40	0.70	0.71	0.73	0.75	0.78	0.81	0.83	0.85	0.87

https://doi.org/10.1371/journal.pmed.1002284.t001

Table 13. Growth chart for fetal femur length/biparietal diameter.

Gestational Age (Weeks)	Femur Length/Head Circumference Ratio by Percentile								
	2.5	5	10	25	50	75	90	95	97.5
14	0.11	0.11	0.11	0.12	0.13	0.14	0.15	0.15	0.16
15	0.12	0.12	0.13	0.14	0.14	0.15	0.16	0.16	0.17
16	0.13	0.14	0.14	0.15	0.15	0.16	0.17	0.17	0.18
17	0.14	0.15	0.15	0.16	0.16	0.17	0.18	0.18	0.19
18	0.15	0.16	0.16	0.17	0.17	0.18	0.19	0.19	0.19
19	0.16	0.16	0.17	0.17	0.18	0.19	0.19	0.20	0.20
20	0.17	0.17	0.17	0.18	0.18	0.19	0.20	0.20	0.20
21	0.17	0.17	0.18	0.18	0.19	0.19	0.20	0.20	0.21
22	0.17	0.18	0.18	0.19	0.19	0.20	0.20	0.21	0.21
23	0.18	0.18	0.18	0.19	0.19	0.20	0.21	0.21	0.21
24	0.18	0.18	0.18	0.19	0.19	0.20	0.21	0.21	0.21
25	0.18	0.18	0.19	0.19	0.20	0.20	0.21	0.21	0.21
26	0.18	0.18	0.19	0.19	0.20	0.20	0.21	0.21	0.22
27	0.18	0.18	0.19	0.19	0.20	0.20	0.21	0.21	0.22
28	0.18	0.18	0.19	0.19	0.20	0.21	0.21	0.22	0.22
29	0.18	0.18	0.19	0.19	0.20	0.21	0.21	0.22	0.22
30	0.18	0.19	0.19	0.20	0.20	0.21	0.21	0.22	0.22
31	0.18	0.19	0.19	0.20	0.20	0.21	0.22	0.22	0.22
32	0.19	0.19	0.19	0.20	0.20	0.21	0.22	0.22	0.23
33	0.19	0.19	0.19	0.20	0.21	0.21	0.22	0.22	0.23
34	0.19	0.19	0.20	0.20	0.21	0.22	0.22	0.23	0.23
35	0.19	0.19	0.20	0.20	0.21	0.22	0.22	0.23	0.23
36	0.19	0.20	0.20	0.21	0.21	0.22	0.23	0.23	0.23
37	0.19	0.20	0.20	0.21	0.22	0.22	0.23	0.23	0.24
38	0.20	0.20	0.20	0.21	0.22	0.22	0.23	0.23	0.24
39	0.20	0.20	0.20	0.21	0.22	0.22	0.23	0.24	0.24
40	0.19	0.20	0.20	0.21	0.22	0.22	0.23	0.24	0.24

https://doi.org/10.1371/journal.pmed.1002284.t002

Reference

1. Kiserud T, Piaggio G, Carroli G, Widmer M, Carvalho J, Neerup Jensen L, et al. (2017) The World Health Organization Fetal Growth Charts: A Multinational Longitudinal Study of Ultrasound Biometric Measurements and Estimated Fetal Weight. PLoS Med 14(1): e1002220. doi:10.1371/journal.pmed.1002220 PMID: 28118360
